# High-Dose Intravenous Vitamin C Combined with Cytotoxic Chemotherapy in Patients with Advanced Cancer: A Phase I-II Clinical Trial

**DOI:** 10.1371/journal.pone.0120228

**Published:** 2015-04-07

**Authors:** L. John Hoffer, Line Robitaille, Robert Zakarian, David Melnychuk, Petr Kavan, Jason Agulnik, Victor Cohen, David Small, Wilson H. Miller

**Affiliations:** 1 Department of Medicine, Jewish General Hospital, Montreal, Canada; 2 Lady Davis Institute for Medical Research, Montreal, Canada; 3 Clinical Research Unit, Segal Cancer Centre, Jewish General Hospital, Montreal, Canada; 4 Department of Oncology, Segal Cancer Centre, Jewish General Hospital, Montreal, Canada; 5 Departments of Oncology and Medicine, Segal Cancer Centre, Montreal, Canada; 6 Departments of Medicine and Oncology, Peter Brojde Lung Cancer Centre, Jewish General Hospital, Montreal, Canada; Cardiff University, UNITED KINGDOM

## Abstract

**Background:**

Biological and some clinical evidence suggest that high-dose intravenous vitamin C (IVC) could increase the effectiveness of cancer chemotherapy. IVC is widely used by integrative and complementary cancer therapists, but rigorous data are lacking as to its safety and which cancers and chemotherapy regimens would be the most promising to investigate in detail.

**Methods and Findings:**

We carried out a phase I-II safety, tolerability, pharmacokinetic and efficacy trial of IVC combined with chemotherapy in patients whose treating oncologist judged that standard-of-care or off-label chemotherapy offered less than a 33% likelihood of a meaningful response. We documented adverse events and toxicity associated with IVC infusions, determined pre- and post-chemotherapy vitamin C and oxalic acid pharmacokinetic profiles, and monitored objective clinical responses, mood and quality of life. Fourteen patients were enrolled. IVC was safe and generally well tolerated, although some patients experienced transient adverse events during or after IVC infusions. The pre- and post-chemotherapy pharmacokinetic profiles suggested that tissue uptake of vitamin C increases after chemotherapy, with no increase in urinary oxalic acid excretion. Three patients with different types of cancer experienced unexpected transient stable disease, increased energy and functional improvement.

**Conclusions:**

Despite IVC’s biological and clinical plausibility, career cancer investigators currently ignore it while integrative cancer therapists use it widely but without reporting the kind of clinical data that is normally gathered in cancer drug development. The present study neither proves nor disproves IVC’s value in cancer therapy, but it provides practical information, and indicates a feasible way to evaluate this plausible but unproven therapy in an academic environment that is currently uninterested in it. If carried out in sufficient numbers, simple studies like this one could identify specific clusters of cancer type, chemotherapy regimen and IVC in which exceptional responses occur frequently enough to justify appropriately focused clinical trials.

**Trial Registration:**

ClinicalTrials.gov NCT01050621

## Introduction

Intravenous vitamin C (IVC) is a widely used alternative cancer treatment [1–3] whose objective of cancer delay, arrest or regression is supported by a large and fairly consistent body of cellular evidence [4–16] and some animal [17–20] and clinical evidence [21–23]. The United States National Cancer Institute provides detailed and up-to-date information about the scientific status of IVC therapy (http://www.cancer.gov/cancertopics/pdq/cam/highdosevitaminc/healthprofessional) even though the website Quackwatch condemns it as a fraud (http://www.quackwatch.org/01QuackeryRelatedTopics/Cancer/c.html). Indeed, despite the common use of IVC by integrative and complementary practitioners [3], there is a serious lack of systematic information pertaining to IVC’s safety and effectiveness in cancer therapy [3,21,22,24–27].

We and others have documented the safety and lack of serious side effects of IVC injections as sole therapy for advanced cancer [26,28,29]. Despite some indication of maintained quality of life when higher doses of the vitamin were infused, we did not observe anti-cancer effects when IVC was used as the sole treatment for advanced incurable cancer that had previously failed all other treatments. The present study was carried out to obtain information about the safety and tolerability of high-dose IVC when combined with cytotoxic chemotherapy. There is biological evidence that high extracellular or tissue concentrations of vitamin C (and other antioxidants) could reduce the toxicity of chemotherapy or increase its efficacy [12,30–33]. However, with the possible exception of adenocarcinoma of the pancreas [21,22] or ovarian cancer [23], no clinical information is available to indicate which cancer types and chemotherapy regimens could be augmented or antagonized by IVC.

This clinical trial had four aims. The first aim was to document the side effects, toxicity and tolerability of IVC in combination with cytotoxic chemotherapy in a consecutive series of patients administered a dose of 1.5 g/kg body weight 2 or 3 times per week. The second aim was to determine the pharmacokinetic profiles of vitamin C and oxalic acid before and after chemotherapy. Chemotherapy and systemic inflammation cause antioxidant depletion, lowering plasma vitamin C concentrations [34–37], potentially increasing formation of vitamin C’s breakdown metabolite, oxalic acid and increasing the risk of calcium oxalate renal stone formation. The third aim was to identify “clusters” of cancer diagnosis and chemotherapy regimen associated with an unexpectedly favorable or unfavorable clinical course. Finally, patients were followed to assess their quality of life and mood while receiving IVC and chemotherapy.

## Methods

### Clinical trial designation and design

This was an early-phase clinical trial whose primary aim was to identify serious adverse events and toxicity related to the combined use of IVC and chemotherapy. The primary aim of phase I clinical trials is to identify a safe and tolerable dose of the investigational drug by administering increasingly large doses to small successive cohorts of volunteer patients, with secondary aims of determining pharmacological and metabolic parameters and screening for indications of clinical benefit [38]. The primary aim of phase II trials is to test for clinical effectiveness using the safe and tolerable dose identified in a phase I trial [39,40]. Phase IIA trials are pilot studies that screen for treatment activity, whereas phase IIB trials are either randomized controlled trials or employ only a single treatment arm which, in principle, should rely on historical or other information as the standard of comparison. The target number of participants is recommended to be determined by the anticipated response based on such prior information. However, some published trials designated as phase I have used only a single dose [22] and some phase II trials set toxicity as their primary aim [39].

The dose of IVC used in this study had been determined in our previous phase I clinical trial of IVC alone [26]. We set toxicity and tolerability as the primary aim despite its record of tolerability in suitably screened people, because there is a dearth of rigorous information about its interactions with standard anti-cancer drugs. The heterogeneity in cancer diagnoses and chemotherapy regimens and the lack of useful historical information in this setting precluded using a statistical model to predict the minimum number of patients to enroll to detect a clinical benefit at a predetermined level of statistical significance. We designate this study as a phase I-II clinical trial, but it could also be described as a single dose phase IIA pilot study [39]. The target enrollment of 24 participants was chosen as the maximum number of patients anticipated to be enrolled within the limitations of time and available funding [40], similar to our earlier, similarly funded phase I trial [26].

Prior to commencing the treatment protocol and after every two chemotherapy treatment cycles (or every two months of continuous therapy, whichever was longer) all the participants were evaluated by computerized tomographic imaging (CT) of the chest, abdomen and pelvis for possible response to treatment, using the Response Evaluation Criteria In Solid Tumors (RECIST 1.0) criteria [41]. Adverse events were evaluated using National Cancer Institute clinical criteria 3.0 [42].

The clinical trial was carried out among patients attending a large medical school-affiliated cancer centre with an attached clinical research unit that specializes in early-phase clinical trials of novel cancer therapies. The eligibility criteria included good functional status (Eastern Cooperative Oncology Group (ECOG) class 0 or 1, normal erythrocyte *glucose-6-phosphate dehydrogenase* activity, a serum creatinine concentration not greater than 175 μmol/L, and the judgment of the treating oncologist that standard-of-care or off-label cytotoxic chemotherapy, while feasible, offered less than a 33% likelihood of an objective clinical response. The main criteria for discontinuation were unacceptable IVC toxicity or disease progression after a minimum of two chemotherapy treatment cycles [41]. The patients’ primary oncologists prescribed the chemotherapy regimen they considered as the most rational in this situation and supervised its administration in the cancer treatment unit. IVC was administered in the nearby clinical research unit. The trial was approved by Health Canada and the Research Ethics Committee of the Jewish General Hospital. The aims, procedures, and potential adverse effects of the study were explained verbally to every eligible and interested patient, who then studied the informed consent document, provided verbal and written consent, and retained a personal copy of the document for reference. After the first patient was successfully enrolled and it was clear that the protocol was feasible, the study was formally registered at clinicaltrials.gov on January 11, 2010 (NCT01050621). The first participant was enrolled March 13, 2009, the second on January 8, 2010, and the final participant completed the protocol on February 18, 2013. Enrollment in the study stopped when the research funding that enabled it was spent. There are no other related or ongoing clinical trials at this center. The full protocol for this trial and supporting TREND checklist are available as supporting information; see S1 TREND Checklist and S1 Protocol.

### IVC infusion protocol

The IVC infusion protocol was previously described [26]; it is based on a well-known protocol developed by Riordan et al [43,44]. Vitamin C infusates were prepared using ascorbic acid 500 mg/mL for injection USP (supplied as single-use 50 mL glass ampules) as a gift from Alveda Pharma Canada, Ltd. The stock solution was diluted in sterile water to achieve an osmolarity of approximately 900 mOsm/L. Any air bubbles formed during preparation were promptly evacuated. The solutions were delivered to the clinical research unit covered by an opaque bag, allowed to come to ambient temperature, and infused by calibrated infusion pump within one hour of preparation. Water and other drinks (preferably sugar-free) were provided and the patients encouraged to consume them freely before, during and after IVC infusions. The dose of vitamin C was 1.5 g/kg body weight when the body mass index (BMI) was 30 kg/m^2^ or less, and normalized to the body weight corresponding to BMI 24 kg/m^2^ for patients with a BMI > 30. The vitamin was infused at a constant rate over a period of 90 minutes for doses up to 90 g, and over a period 120 minutes for doses > 90 g. IVC was infused three times (at least one day apart) on week days during weeks when chemotherapy was administered (but not on the same day as intravenous chemotherapy) and any two days at least one day apart during weeks when no chemotherapy was given.

### Pharmacokinetic studies

Less than 7 days before the first chemotherapy dose, participants were administered a sub-maximal dose of vitamin C (0.6 g/kg of ascorbic acid) by continuous infusion over a period of 90 minutes. Plasma vitamin C concentrations and vitamin C and oxalic acid urinary excretion were measured during the infusion and over the following 4 hours as previously described [26,45]. The same study was repeated approximately 3 days after chemotherapy administration. These studies were carried out in 12 of the 14 participants. The dose of 0.6 g/kg was chosen to allow sensitive detection of any reduction in peak vitamin C concentration or reduced urinary excretion, which would indicate any increase of vitamin C cellular uptake or increased catabolism caused by the chemotherapeutic agent.

### Monitoring

Upon study entry and at the beginning of every 4 week cycle, patients underwent a physical examination and laboratory evaluations that included a complete blood count, serum chemistries, the coagulation profile, the inflammation marker C-reactive protein (CRP), and the following tumour markers when appropriate: carcinoembryonic antigen (CEA), cancer antigen 125 (CA-125), cancer antigen 15–3 (CA-15-3). CT examinations were performed for staging and tumour response within 4 weeks prior to the first IVC infusion and after approximately every second chemotherapy cycle. Participants completed the FACT-G quality of life (Functional Assessment of Cancer Therapy—General) questionnaire, in which scores range from 0 to 108 [26]. The total mood disturbance (TMD) score of the Profile of Mood States-B questionnaire [37] was used to assess mood state (scores range from -20 to 100; higher values indicate greater mood disturbance). These questionnaires were administered at baseline, after 2 weeks on protocol and approximately monthly thereafter. Toxicity assessments were performed continuously while on the trial. Every patient was followed by his or her own oncologist, the nurses in the oncology treatment unit, the nurses in the clinical research unit, and the investigators. The duration of known stable disease was defined as the number of days from the first IVC infusion to the last CT scan that documented stable disease.

## Results

**Fig 1:**
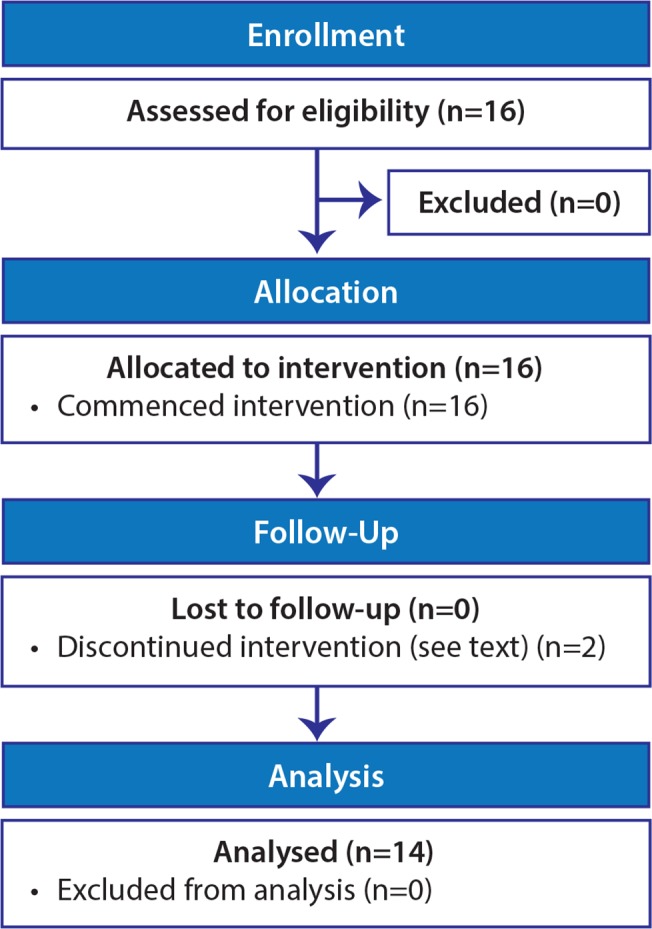
Flow diagram.

In addition to the cases described below, 17 patients were brought to our attention as interested and potentially eligible but ultimately were not formally evaluated nor enrolled because their clinical condition deteriorated, they opted to participate in another clinical trial, decided that participation would be too time consuming, or for other reasons. No further information was obtained about these patients since they did not participate in the clinical trial. As shown in the flow diagram (Fig 1) two patients were formally enrolled in the protocol but their condition deteriorated before IVC therapy could get underway. Consequently, no relevant clinical information was obtained about these patients. The fourteen patients who participated in the protocol are the subject of this report (Fig 1). Their characteristics are shown in Table 1, and the details of their treatment are shown in Table 2. The heterogeneity of diagnoses, treatments, and clinical courses was too great to make a simple summary table of responses meaningful.

**Table 1:** Patient characteristics at study enrollment.

ID	Age	Sex	Diagnosis	Previous therapy	ECOG	FACT-G	TMD
1	73	F	Lung	None	0	100	-6
2	57	M	Colon	Surgery, chemotherapy	1	65	22
3	71	M	Colon	Surgery, chemotherapy	0	73	2
4	52	F	Rectum	Surgery, chemotherapy, radio- and brachytherapy	1	76	9
5	58	F	Rectum	Surgery, radiotherapy, chemotherapy	0	86	-9
6	55	M	Rectum	Surgery, chemotherapy	1	76	13
7	76	M	Colon	Surgery, chemotherapy	0	72	16
8	63	M	Bladder	Surgery, chemotherapy	1	77	33
9	67	F	Ovary	Surgery, chemotherapy	0	55	38
10	47	F	Cervix	Chemotherapy, radio- and brachytherapy	0	43	34
11	54	F	Biliary tract	Chemotherapy	1	59	21
12	54	F	Breast	Surgery, radiotherapy, Chemotherapy	0	72	16
13	63	M	Tonsil	Chemotherapy	1	68	6
14	58	M	Lung	Chemotherapy, radiotherapy	1	84	0

**Table 2:** Metabolic and treatment details.

ID	Wt (kg)	BMI (kg/m^2^)	Plasma vitamin C (μmol/L)*	Serum creatinine (μmol/L)	Serum CRP (mg/L)	Serum albumin (g/L)	IVC dose (g)	Treatment duration (days)	Number of IVC infusions
1	64	26	47	60	4.2	45	90	85	28
2	80	25	9.5	66	148	44	124	34	15
3	73	27	35	94	4.9	42	110	72	26
4	77	25	29	45	66.5	40	115	41	13
5	69	27	84	69	9.6	43	103	62	24
6	107	34	28	80	32	41	112	114	37
7	100	32	47	83	2.8	42	112	115	40
8	88	28	292	72	34.5	36	108	12	6
9	73	30	36	90	15.1	41	108	83	24
10	75	30	56	80	10.2	40	112	193	57
11	58	23	38	63	7.9	43	88	580	173
12	68	25	96	42	1	41	102	46	15
13	57	20	-	63	8.0	44	86	220	65
14	64	23	-	45	30.7	40	96	11	6

* A value < 28.4 μmol/L indicates hypovitaminosis C.

### Adverse effects and toxicity

IVC was nontoxic for all participants. Thirst and increased urinary flow were predictable and common minor symptoms during all IVC infusions. Three patients experienced notably unpleasant side effects: nausea and occasional vomiting during the IVC infusion (patient 4); thirst and an unpleasant fluttering sensation in the upper abdomen during the IVC infusion and a mentally hazy feeling the day after it (patient 7); chills, thirst, headache, and a rumbling feeling during the IVC infusion, as well as increased leg edema for a few days after each infusion (patient 10).

### Pharmacokinetics

**Fig 2:**
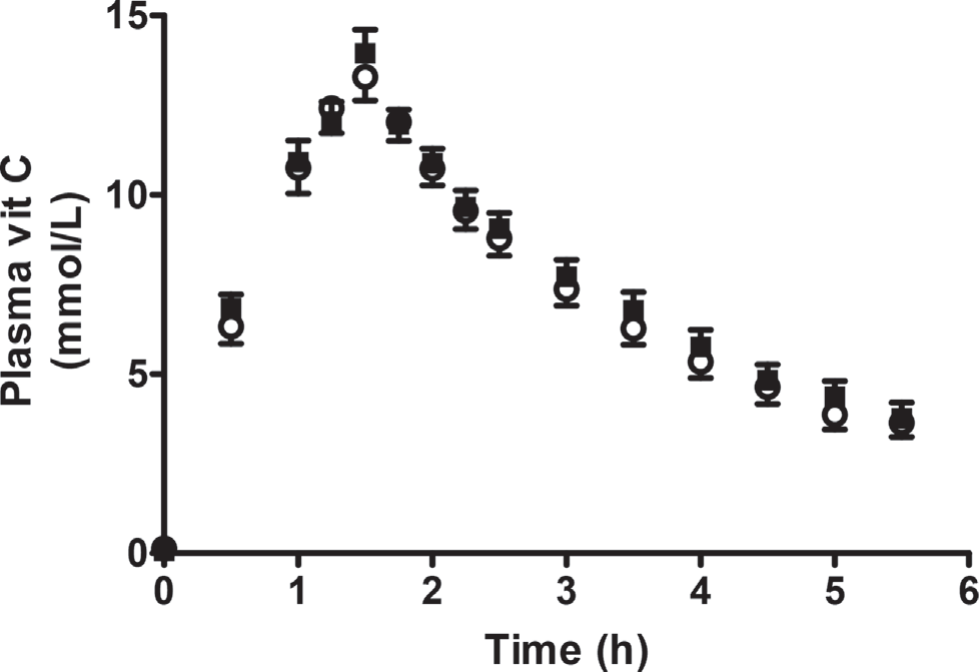
Mean plasma vitamin C concentrations ± SEM (N = 12) during and following infusion of 0.6 g/kg vitamin C, before (■) and after (○) chemotherapy. AUC (areas under the curve) were not significantly different (p = 0.146, paired t-test).

As shown in Table 2, only two patients in this trial (patients 2 and 6) had hypovitaminosis vitamin C when they entered the study. Pre- and post-chemotherapy plasma and urinary vitamin C and urinary oxalic acid pharmacokinetic profiles were obtained in 12 of the 14 participants (see Fig 1 and Table 3). The plasma vitamin C concentration-time profile was not affected by chemotherapy (Fig 2), but the urinary excretion profiles suggested at least short-term tissue retention of the vitamin following chemotherapy with no increase of urinary oxalic acid excretion. Urinary oxalic acid excretion represented a trivial proportion of the infused dose both before and after chemotherapy. The individual values for vitamin C and oxalic acid concentrations and vitamin C time-course profiles are available as supporting information; see S1 Text.

**Table 3:** Pharmacokinetic profiles before and after chemotherapy.

Parameter	Before	After	P**
Vitamin C dose infused (g)	43 ± 4	43 ± 4	
Vitamin C dose (mmol)	243 ± 24	243 ± 24	
Baseline plasma concentration (μmol/L)	66.4 ± 74.9	131.6 ± 102.0	0.031
Plasma concentration end of infusion (mmol/L)	14.0 ± 2.3	13.5 ± 2.2	0.278
Plasma concentration 4 h later (mmol/L)	3.8 ± 1.5	3.6 ± 1.4	0.784
Vitamin excretion by end of infusion (mmol)	51.4 ± 22.8	39.2 ± 16.4	0.041
(% of dose)	(21.4 ± 9.5)	(16.6 ± 7.6)	
Total excretion by 4 h after end of infusion (mmol)	149 ± 49.3	133 ± 40.0	0.099
(% of dose)	(62.2 ± 20.7)	(55.1 ± 16.5)	
Oxalic acid excretion by end of infusion (mg)	10.9 ± 6.8	12.3 ± 9.6	0.791
Total oxalic acid excreted 4 h after end of infusion (mg)	40.7 ± 19.6	40.9 ± 20.1	0.850
(% of vitamin C dose*)	(0.19 ± 0.09)	(0.19 ± 0.10)	

The data are presented as mean ± SD.

* Moles of oxalic acid excreted per 100 mol of vitamin C administered.

** Wilcoxon matched pairs test (GraphPad Prism version 5.01).

Analysis of the individual data confirmed the accuracy of a previous empirically developed formula [26] for predicting the post-infusion peak plasma vitamin C concentration in a patient with normal renal function, namely, plasma vitamin C concentration (g/L) = 3.75 D/W, where D is the ascorbic acid dose in g, and W is body weight in kg. (To convert g/L to mmol/L, multiply by 5.7. For example, a patient with body weight 70 kg infused 105 g vitamin C will have a peak plasma vitamin C concentration of 5.6 g/L = 32.0 mmol/L.)

### Clinical responses

Two patients who enrolled in the study deteriorated so rapidly, in ways that could not reasonably be attributed to IVC therapy, that no conclusion about its benefit or harm was possible (patients 8 and 14). Six patients experienced neither objective nor subjective benefit as a result of their participation in the clinical trial (patients 2, 3, 4, 5, 9, 12). Four of them had advanced colorectal cancer (patients 2, 3, 4, 5); the others had cancer of the ovary (9) and breast (12). Six patients experienced either transient stable disease (patients 1, 6, 7) or longer-lasting but impermanent stable disease (10, 11, 13).

Three patients (patients 10, 11, 13) had unusually favorable experiences that were deemed highly unlikely to result from chemotherapy alone (probability much less than 33%). One patient had transient stable disease with increased energy and strong functional improvement with a marked reduction in tumor-related leg edema even though her chemotherapy consisted merely of infrequent infusions of a drug that had previously been found to be ineffective when given much more frequently (patient 10). A second patient experienced stable disease for approximately 14 months despite having cancer of the ampulla of Vater, a chemotherapy-resistant cancer that had earlier progressed during empirical chemotherapy (patient 11). The third patient experienced stable disease and improved well-being even though chemotherapy had been deemed highly unlikely to be beneficial (patient 13). In order to portray the entire clinical outcome of this trial, the experience of every participant is summarized in the following paragraphs. More detailed individual case information is available as supporting information; see S2 Text.

Case 1. At age 73 this previously healthy woman was found to have asymptomatic stage IV adenocarcinoma of the lung. She received IVC plus standard first-line chemotherapy (carboplatin and docetaxel in 3-week cycles). Stable disease was documented after the second treatment cycle. After the fourth cycle the original target lesions remained stable but a new nodule of unstated size was detected in the posterior apex of the right lung. The oncologist determined that this new lesion was proof of cancer progression and the protocol was terminated. Apart from the side effects of her chemotherapy, the patient remained well with normal quality of life. She experienced no adverse events attributable to vitamin C. Her CRP level remained normal and constant. During the period of IVC infusion the FACT-G score was constant. The TMD score improved from -6 to -14. Duration of stable disease: 46 days. IVC duration: 85 days (28 infusions).

This patient with advanced lung cancer had stable disease after 2 cycles of chemotherapy, but after 2 more cycles there was evidence of progression in the form of a small new lung mass. Patients with stage IV lung cancer usually experience progressive disease after first-line chemotherapy. This patient’s brief period of stable disease could have been due to her chemotherapy, the combination of chemotherapy and IVC, or the natural history of her disease.

Case 2. At age 54 this man was found to have colon cancer with spread to his abdominal and pelvic lymph nodes, liver and lungs. After resection of the primary lesion he received adjuvant chemotherapy followed by several different chemotherapy regimens, including irinotecan, but none of them halted the cancer’s progression. He enrolled in the IVC trial and was treated with irinotecan, fluorouracil, folinic acid and bevacizumab at 2 week intervals. After 6 weeks of treatment bilateral leg swelling developed and a CT scan showed disease progression. The protocol was discontinued. His serum CRP concentration, which was markedly increased at trial entry (148 mg/L), decreased moderately during treatment on the protocol. The FACT-G was constant and TMD score improved from 22 to 16. IVC duration: 34 days (15 infusions).

IVC and chemotherapy were administered for 34 days without toxicity, side effects or benefit.

Case 3. At age 68 this man was found to have an invasive well-differentiated adenocarcinoma of the colon with spread to regional lymph nodes. A right hemicolectomy was performed. The cancer progressed despite 3 years of chemotherapy. He enrolled in the protocol, which included two-week cycles of irinotecan, fluorouracil and folinic acid. After the fifth cycle a CT scan indicated progression of all the intra-abdominal implants. The protocol was discontinued. The serum CEA concentration, which was approximately 130 ng/mL at protocol entry, increased to approximately 200 ng/mL during combined chemotherapy and IVC. The CRP concentration was normal on trial entry and remained so. The FACT-G score was constant. The TMD score improved from 2 to -8. IVC duration: 72 days (26 infusions).

IVC and chemotherapy were administered for 72 days without toxicity, side effects or benefit.

Case 4. This woman was diagnosed with rectal cancer at age 45 with residual cancer present following surgery. She received chemotherapy and radiotherapy. Five years later the cancer recurred locally and in the form of a pre-sacral tumour mass with involvement of the cervix. There was no response to two different chemotherapy regimens and local brachytherapy. She enrolled in the IVC trial. Chemotherapy on protocol consisted of two weeks of daily oral capecitabine followed by a one week rest period. Early during treatment the patient volunteered that she experienced increased energy after IVC infusions, but later on began to experience nausea and occasional vomiting near the end of the infusions. The nausea was unrelieved either by restricting her consumption of sugary drinks during the infusions or reducing the IVC dose, and she had to withdraw from the study. A CT scan carried out 16 days after study withdrawal indicated that a previously identified target nodule in the right upper lobe of her lung had increased in size, evidence of disease progression. Her serum CRP remained high and approximately constant throughout the course of treatment. The FACT-G score did not change. The TMD score worsened from 9 to 22. IVC duration: 41 days (13 infusions).

Despite initially feeling increased energy following IVC infusions, this patient ultimately withdrew from the trial because the infusions induced nausea and occasional vomiting. There was no benefit from IVC and chemotherapy.

Case 5. This woman was found to have rectal cancer at age 56. She underwent a hemicolectomy followed by chemoradiation therapy. The disease recurred a year later and further surgery was carried out. However, three months afterwards the cancer was found to have spread to her liver and lungs, and it continued to progress despite two different chemotherapy regimens. The patient enrolled in the IVC trial, taking oral capecitabine tablets daily for 2 weeks followed by a one week rest period. At the time of enrollment the CT scan showed liver and bilateral pulmonary nodules; the serum CEA concentration was 27 ng/mL. After 2 months on the protocol a CT scan showed an increased size of the lung and liver nodules; the serum CEA concentration doubled. The CRP remained normal. Disease progression was diagnosed and the protocol was discontinued. The FACT-G score was constant. The TMD score worsened from -9 to 3. IVC duration: 62 days (24 infusions).

This patient’s rectal cancer had progressed despite many previous chemotherapy regimens, and it continued to progress despite IVC and chemotherapy.

Case 6. This man was found at age 53 to have an invasive, moderately differentiated adenocarcinoma of the rectum with spread to his regional lymph nodes, liver and lungs, treated by hemicolectomy and chemotherapy. The cancer progressed despite two different chemotherapy regimens. The patient enrolled in the IVC protocol with irinotecan administered at 3 week intervals. The baseline CT scan indicated many metastatic lesions in his lungs, liver and abdominal lymph nodes. The serum CEA concentration was 532 ng/mL. After 4 cycles of chemotherapy all the lesions had increased in size, but by less than 20% and hence did not meet the criteria for disease progression. Further CT imaging 6 weeks later definitely showed progression and identified new brain metastases. The protocol was discontinued. During treatment the serum CRP concentration increased from a baseline of 30 mg/L to 120 mg/L. The CEA concentration approximately doubled. The FACT-G score decreased from 76 to 66. The TMD score improved from 13 to -1. Duration of known stable disease: 44 days. IVC duration: 114 days (37 infusions).

This patient with advanced rectal carcinoma unresponsive to previous chemotherapy received IVC and chemotherapy without adverse events, toxicity or benefit.

Case 7. This man was found at age 56 to have colon cancer with spread to his liver and lungs. Despite four different chemotherapy regimens over the next 3 years the cancer ultimately progressed. Upon enrollment in the IVC trial he received 3-week cycles of irinotecan, bevacizumab and capecitabine. The serum CEA concentration was 359 ng/mL; the CRP concentration was normal. The IVC infusions produced epigastric discomfort and a fluttering feeling in his upper abdomen, occasionally bordering on nausea, during the final minutes of every infusion. Additionally, he experienced fatigue and a hazy mental feeling which persisted over the day following an infusion. He experienced stable disease for 3.5 months, at which time a CT scan indicated increased size of one liver mass. The protocol was terminated. During the period of stable disease the serum CEA concentration doubled. The FACT-G score was constant. The TMD score varied without a directional change. (This patient subsequently experienced an important biochemical and clinical remission when treated with the biologic agent, panitumumab.) Duration of known stable disease: 54 days. IVC duration: 115 days (40 infusions).

This patient with progressive chemotherapy-resistant colon cancer experienced unpleasant but tolerable side effects during IVC and chemotherapy with transient stable disease.

Case 8. This 61 year old man underwent a radical cystectomy with creation of an ileal urinary conduit for high grade bladder carcinoma. At surgery he was found to have direct extension of the cancer beyond the bladder with perineural invasion and regional lymph node involvement. He received many different chemotherapy regimens without benefit, and was enrolled in the IVC trial. At the time of enrollment he was taking approximately 30 g/day vitamin C by mouth, and his plasma vitamin C concentration reflected this (see Table 2). The chemotherapy regimen consisted of 3-week cycles of gemcitabine and cisplatin. However, after the first dose of chemotherapy and 4 therapeutic IVC infusions he developed fever, confusion and hypotension from gram-negative sepsis. Chemotherapy and IVC infusions were stopped and the protocol was terminated. IVC duration: 12 days (6 infusions).

No conclusion about IVC and chemotherapy can be drawn from this case.

Case 9. At age 65 this woman was found to have cancer of the left ovary with peritoneal involvement and ascites. Her cancer progressed despite neo-adjuvant chemotherapy, repeated surgical interventions and different chemotherapy regimens. She was enrolled in the IVC protocol, which involved carboplatin infusions at 3 week intervals. The IVC infusions caused thirst and shakiness that were mitigated by slowing the infusion rate. After 3 cycles of carboplatin her large lower abdominal mass increased in size, a clear clinical indication that her disease was progressing. The protocol was terminated. There was no change in quality of life. During this time the CA-125 concentration approximately doubled. The CRP concentration decreased from 32 mg/L to approximately 15 mg/L. FACT-G and TMD scores were inadequately obtained for this patient. IVC duration: 83 days (24 infusions).

This 67 year old woman with advanced chemotherapy-resistant ovarian cancer experienced mild side effects and no benefit from IVC and chemotherapy.

Case 10. This woman was diagnosed with poorly differentiated stage II B adenocarcinoma of the cervix at age 43. Several cycles of cisplatin, external radiotherapy and brachytherapy brought about clinical remission, but the disease recurred two years later and hospice care was recommended to her. Instead she came to this hospital where treatment with carboplatin and paclitaxel led to a dramatic clinical improvement with virtual disappearance of disabling bilateral leg edema due to intrapelvic lymph node invasion by the cancer. Carboplatin therapy continued (with carboplatin desensitization after a severe allergy to it developed) but the patient was unable to tolerate the physical and mental disability it caused, so the regimen was changed to paclitaxel at weekly intervals. On this therapy the cancer progressed. The patient enrolled in the IVC protocol. Chemotherapy consisted simply of paclitaxel infused at three week intervals. She also took 10,000 IU per day of vitamin D. After 4 weeks on the protocol the patient experienced a marked improvement in her energy level that lasted for a few days after each IVC infusion. Her feeling of well being and increased energy occurred despite unpleasant symptoms of chilliness, thirst, headache, rumbling feelings and shakiness while the IVC infusion was in progress and despite an increase of her right leg edema after each infusion that required several days to regress. She contrasted the feeling of well being associated with IVC infusions to the “bloated-fatigued” feeling that had become the normal condition of her life with her disease. The symptoms of shakiness, headache and thirst were eliminated by slowing the IVC infusion rate. Stable disease was documented after two months on the protocol despite previous progression when the same chemotherapy was given much more frequently. At this point the patient chose to discontinue the paclitaxel injections entirely and requested IVC therapy alone; the request was granted. The patient continued to feel well with IVC therapy, stating that she felt globally better than at any time in the last 4 years. Marked right leg edema had been her most prominent physical symptom, and over the course of protocol it decreased in volume by approximately one half. For the first time in several years she was able to walk 1 km/day and attend her local gym to go swimming. She attributed her sense of well-being to the IVC infusions and continued to refuse chemotherapy. After a total 175 days of IVC therapy a CT scan showed local disease progression. Two weeks later she suddenly experienced twitching of her right arm and face. A CT scan of the head showed a left frontal cortical mass. All anti-cancer therapy treatment, including vitamin C, was stopped, and she entered hospice care. Upon study enrollment the serum CA-125 concentration was 230 U/mL. The concentration steadily increased during IVC and chemotherapy to a maximum of 1030 U/mL. The FACT-G score improved during IVC. The TMD score varied widely without a directional trend. Duration of known stable disease: 84 days. IVC duration: 193 days (57 infusions).

This 48 year old woman had advanced poorly differentiated cancer of the cervix that progressed despite paclitaxel infusions every 7 days but was stable when IVC was infused together with paclitaxel administered only every 3 weeks, with improved quality of life. The improved quality of life may partly have been due to simple avoidance of the side effects of chemotherapy. Subsequent IVC alone did not arrest disease progression.

Case 11. At age 54 this woman required emergency placement of a biliary stent and cholecystostomy tube to treat biliary tract obstruction by adenocarcinoma of the ampulla of Vater with multiple liver metastases. Five cycles of intravenous gemcitabine and oral capecitabine failed to halt the cancer’s progression. The patient enrolled in the IVC trial, receiving 3 week cycles of intravenous oxaliplatin and oral capecitabine. The baseline CT scan showed innumerable scattered hypodensities throughout the liver. The serum CEA concentration was 30 ng/mL. The patient maintained good functional status and stable disease for 17 months, at which time a CT scan indicated a small new lung nodule, worsening bile duct dilation, and enlargement of a previously stable liver nodule. The CEA concentration remained constant and the CRP level remained normal or near-normal. The extraordinary period of time this patient had enjoyed stable disease encouraged her oncologist to continue the IVC while he considered an alternative chemotherapy regimen, so IVC therapy continued twice weekly for 3 more months, at which time the oncologist concluded that he could not scientifically justify any other chemotherapy regimen, and the protocol was terminated. A few days later the patient suffered a fall and was found to have a large metastatic lesion involving the C2 vertebral body. She was admitted to hospital for palliative radiation and hospice care. During her time on the IVC protocol the FACT-G score did not change. The TMD score fluctuated without any directional trend. Duration of known stable disease 397 days. IVC duration: 580 days (173 infusions).

While receiving IVC and chemotherapy this patient experienced an unexpected and unprecedentedly long period of stable disease with maintained quality of life despite a previously progressive chemotherapy-resistant cancer of the ampulla of Vater.

Case 12. At age 50 this woman was found to have right breast cancer treated with several cycles of neoadjuvant chemotherapy, surgery, radiotherapy and tamoxifen. The cancer recurred a year later, and it progressed despite treatment with several anti-cancer drugs She enrolled in the IVC protocol, receiving capecitabine and trastuzumab every 2 weeks. Her CA 125 and CA 15–3 concentrations, already high at baseline, increased further on the protocol. She experienced no adverse effects of toxicity attributable to the IVC, but found the time commitment and inconvenience of continued participation too onerous, and withdrew from the study. Her FACT-G score did not change, and the TMD score varied without any net change. IVC duration: 46 days (15 infusions).

This patient experienced neither adverse effects nor benefit from IVC and chemotherapy.

Case 13. At age 63 this man was found to have a poorly differentiated epidermoid carcinoma involving his tonsils, with invasion of the base of the skull, paranasal and sphenoid sinuses and chest. Radiotherapy and 3 cycles of carboplatin and fluorouracil eliminated all these lesions, but the disease recurred 3 years later. His oncologists told him that futher chemotherapy was futile. He instead came to our institution and was enrolled in the IVC protocol, receiving carboplatin and docetaxel in 3 week cycles. The baseline CT scan showed a large destructive mass in the left nasal cavity with destruction of the medial wall of the left maxillary sinus and nasal turbinates and extension into the sphenoid sinus, destroying the floor of the left orbit and extending into his left maxillary bone, parapharyngeal soft tissues and left hard palate. There was a large metastatic lesion in the left upper lobe of his lung. Within 2 months on the protocol there was clear-cut improvement in hearing in his left ear. His mostly occluded left nasal passage became free, allowing him to sleep normally for the first time, and he could open his jaw more fully. After three more cycles a CT indicated stable disease. Approximately 3 cycles after that, he reported that hearing acuity in his left ear had worsened again, although he also experienced decreasing numbness in his left jaw. The CT scan showed decreased overall size of the extensive tumor mass that was invading his sinuses and the base of his skull, but since the decrease in size was < 15%, his status was evaluated as stable disease. The patient felt very well, exercised frequently, and decided to take a two-week vacation that required discontinuing the IVC and chemotherapy. During the final days of the vacation he sensed increasing pressure in his hard palate. A CT scan carried out upon his return indicated increased size of the tumor mass. Even though the increase in size was less than 30% and hence classified as stable disease, the general picture was one of disease progression. It was decided to continue the IVC and change the chemotherapy to gemcitabine and capecitabine. After two cycles the tumor progressed further, and the protocol was terminated. During his time on the protocol the FACT-G score improved from 66 to 83. The TMD score improved from 6 to -1. The CRP concentration was normal or near-normal throughout. Duration of stable disease 112 days. IVC duration: 220 days (65 infusions).

Despite a nearly hopeless prognosis this patient experienced a temporary but important symptomatic and functional improvement with temporary stable disease during treatment with IVC and chemotherapy.

Case 14. This man was diagnosed at age 53 with small cell carcinoma of the lung with a brain metastasis which initially responded to chemotherapy and radiation therapy to the chest and brain, but the cancer recurred within 3 years. He enrolled in the IVC trial with the plan to administer paclitaxel every second week. Despite concern that the patient’s dyspnea might be worsened by the hyperosmolar IVC infusion, treatment proceeded. Careful monitoring indicated no increase in dyspnea, and oxygen saturation was unchanged during the IVC infusions. Despite treatment, his dyspnea steadily worsened to the point that the protocol was terminated. IVC duration: 11 days (6 infusions).

This patient was enrolled at an advanced stage of disease, and his clinical condition continued to worsen during the two weeks he was on the protocol. High-dose IVC and chemotherapy did not worsen his dyspnea.

## Discussion

This was a consecutive case phase I-II clinical trial of IVC combined with cytotoxic chemotherapy in patients whose treating oncologist judged that standard-of-care or off-label chemotherapy offered less than a 33% likelihood of a meaningful objective response.

We and others have documented IVC’s safety and lack of serious side effects when administered as the sole therapy for advanced chemotherapy-resistant cancers, but also its lack of an objective anti-cancer effect as determined according to RECIST guidelines [26,28]. On the other hand, recent preliminary evidence suggests that the addition of IVC to conventional chemotherapy could increase its efficacy or reduce its toxicity [21–23,29].

The patients enrolled in this clinical trial could differ importantly from patients participating in trials carried out in an integrative, complementary or alternative medicine setting. They had typically undergone several previous treatments including off-label conventional therapy. We registered the experience of every patient who was enrolled to avoid the bias of cherry-picking more interesting cases. A number of patients were brought to our attention as interested and potentially eligible but ultimately did not participate. Patients with advanced incurable cancers accept or refuse enrollment in early-phase clinical trials for many reasons [46,47]. The decision process is even more complicated in the case of complementary, integrative or alternative therapy [48].

This study indicates that combination IVC-chemotherapy is non-toxic and generally well tolerated when safeguards and mitigating procedures are used. Adverse events may nevertheless occur. These adverse events, pre- and post-chemotherapy vitamin C kinetics, and the participants’ clinical responses are discussed in the following paragraphs.

### Adverse effects and toxicity

The adverse symptoms associated with high-dose IVC infusions do not appear to be caused by the ascorbate molecule, but instead by the temperature of the infused solution, its osmolarity, and the sodium load delivered. Any solution rapidly infused at room temperature may cause chills, a problem that can be mitigated by explaining the situation to the patient, allowing the solution to be close to room temperature when infused, and adjusting the infusion volume and rate within reasonable parameters. Any hyperosmolar solution will induce thirst and cause a solute urinary diuresis associated with feelings of shakiness, pressure or discomfort. These symptoms can be prevented or mitigated by an appropriate adjustment of the dose and/or infusion rate. Symptoms are much milder when the patient starts out well-hydrated and remains so during the infusion. Frequent visits to the bathroom are expected and unavoidable. The sodium load imposed by IVC infusions can create a problem. Caution or avoidance may be necessary in patients with extracellular fluid sequestration, as occurs when venous return from a limb is restricted or in the presence of entrapped third space accumulations such as pleural or pericardial effusions. One patient with advanced lung cancer and dyspnea received IVC with careful monitoring without any drop in oxygen saturation or worsening of his dyspnea. Non-entrapped third space accumulations do not necessarily represent a problem. One patient with advanced gastric cancer and extremely distressing ascites was admitted to hospital for hospice care. Although he was ineligible for enrollment in the clinical trial, he and his family urgently requested off-trial IVC therapy on compassionate grounds. IVC smoothly eliminated the ascites and greatly improved his quality of life.

In humans, vitamin C and its metabolites are eliminated only by the urinary route. Consequently, high-dose IVC is contra-indicated when renal function is compromised [45].

Finally, it should be noted that high plasma vitamin C concentrations interfere with most skin-prick or blood glucose measurements by generating either a falsely high or falsely low result [49,50]. For this reason, it is important not to measure (or if inadvertently measured, discount) the blood glucose concentration indicated by such tests until 12 hours after an IVC infusion has ended. If necessary, serum glucose can be accurately measured using the *hexokinase* method.

### Pharamacokinetic profile

Pharmacokinetic studies were carried out to determine whether the pro-oxidant effect of chemotherapy alters vitamin C’s pharmacokinetic profile and increases formation and urinary excretion of its breakdown metabolite, oxalic acid. Chemotherapy had no effect on the plasma vitamin C time profile, but a greater fraction of the dose appeared to be retained within the tissues (at least temporarily) following chemotherapy, with no increase in urinary excretion of the breakdown metabolite, oxalic acid (see Table 3). Since ascorbic acid breakdown to oxalic acid can increase the risk of renal stone, this finding addresses an important safety concern regarding the concurrent use of IVC and chemotherapy.

We verified the accuracy of a previously described vitamin C dose selection equation for achieving peak plasma concentrations in patients with normal renal function [26]. A formula of this kind is useful because many IVC trials base the dose on a target post-infusion plasma vitamin C concentration [22].

### Objective anti-cancer effects

Eligibility for this clinical trial was contingent on the treating oncologist’s judgment that there was a less than 33% likelihood that chemotherapy alone would lead to temporary stable disease or regression. Six of the 12 patients whose response could be evaluated experienced either brief or longer lasting disease stabilization with symptomatic improvement. Three cases, in which the likelihood of a response was judged to be very much less than 33%, had unusually favorable clinical trajectories that could represent exceptional responses or “index cases” for future studies that focus on these cancer types (cervix, biliary tract, and head and neck). On the other hand, 6 patients with colorectal cancer had disappointing experiences, suggesting that chemotherapy-resistant advanced colorectal cancer may be a poor target for future clinical trials of IVC plus conventional chemotherapy.

### Quality of life and mood

It is a common anecdotal experience that IVC produces a boost in energy or well being, and some formal evidence supports the possibility [51–54]. The report of a boost in energy by 3 of the participants in this trial echoes similar reports by patients in our earlier clinical trial [26]. Not all patients had this experience, however. The quality of life and mood questionnaires used in this study would not capture this effect, since they were administered at intervals of weeks, whereas the energy boost associated with IVC appeared to last for only a few days after each vitamin infusion. Beneficial effects of IVC on mood and energy level may be unrelated to its potential anti-cancer effects. Two clinical trials reported improved quality of life [52] or mood [54] with IVC therapy that involved doses of 10 g or less.

### Strengths and weaknesses

This study has important weaknesses inherent in phase I clinical trials, including a low enrollment rate. The patients referred by their oncologists for participation represented a very small fraction of the potentially eligible ones treated in this large cancer treatment center. With one exception (patient 1, who received IVC together with first-line chemotherapy for lung cancer), all of them had very advanced disease that had failed several other treatments. Consequently, the patients enrolled in the study cannot be considered representative of all patients with chemotherapy-resistant cancers. Another weakness is the absence of a control group that would provide assurance that the unusually favorable clinical trajectories of three of the patients is attributable to the addition of IVC to their chemotherapy. No such assurance can be given, other than the clinical judgment of their oncologists that the improvement they experienced had a probability that was much less than 33%. Statistically improbable responses to ineffective chemotherapy do occur—it is for precisely this reason that off-label, ineffective and unproven chemotherapy is very common in conventional cancer care. A final weakness is the fact that disease progression was the protocol-terminating event. The study did not assess long-term survival or long-term quality of life. These are what matter most to patients, but their assessment would require a different and more costly clinical trial design. Thus, this study was not designed either to prove or disprove that the addition of IVC to conventional chemotherapy improves clinical outcomes in cancers of biliary tract, cervix and head and neck, but it did identify these cancer types as potential future clinical targets.

The study has strengths. It provides useful clinical information about the safety and side effects of IVC and information about its pharmacokinetic profile. In agreement with other reports, our results suggest that IVC is acceptably safe and that it does not necessarily block the beneficial effects of chemotherapy [23,29,32,55,56], justifying its continued use in clinical trials [21–23]. Finally, even though this study does not in itself provide evidence that the addition of IVC to conventional chemotherapy will improve the clinical outcomes of patients with cancers of the biliary tract, cervix or head and neck, it does suggest that similar exceptionally favorable responses by other patients with these kinds of cancer would be important to know about. Any role for IVC in cancer therapy, if there indeed is one, will ultimately emerge from new basic understanding of cancer biology [57]. Pending such discoveries, and given IVC’s widespread use by integrative and complementary cancer therapists, our study protocol illustrates the potential value of an individual case-centered evaluation strategy called “discovery in clinical practice” that has been advocated for discovering new indications for conventional drug therapies [58]. Our study demonstrates that it is feasible and relatively inexpensive to objectively document clinical responses with the aim of identifying specific clusters of cancer type, chemotherapy and an appropriate IVC regimen in which unusually favorable clinical courses are over-represented. If identified, such clusters could become the focus of future, definitive clinical trials.

## Conclusions

A chief interest of this early-phase cancer trial for general readers is the paradoxical nature of IVC therapy. Despite its biological and clinical plausibility, with only rare exceptions [11] it is ignored by conventional cancer investigators and funding agencies even as integrative and complementary cancer therapists prescribe it widely, without reporting the kind of clinical data that is normally gathered in conventional cancer drug development [27].

The present state of cancer chemotherapy is unsatisfactory. New cancer drugs continue to be developed and approved on the basis of marginal improvements in survival at an unsustainably high financial cost [59]. It would seem more rational for cancer investigators to attempt to improve the effectiveness of well known, inexpensive generic cancer chemotherapies by studying their clinical interactions with antioxidants, especially vitamin C [32]. However, the lack of financial reward and tainted association with alternative medicine could dissuade conventional investigators and funding agencies from seriously considering this approach. At present, the few cancer clinical trials of IVC being carried out are, like this one, small and supported by limited funding from integrative cancer foundations. Even if serious interest in funding and carrying out large, formal clinical trials were to develop, data are lacking as to which cancers and chemotherapy regimens to focus on.

The present study neither proves nor disproves IVC’s value in cancer therapy. The present data indicate it would be premature to attempt unfocused phase III clinical trials of this therapy at the present time. This study does provide useful information, and suggests a feasible, individual-case oriented strategy for evaluating plausible but poorly understood and unproven metabolic therapies in a mainstream academic environment that is uninterested in them [60]. If carried out in sufficient numbers, simple studies like this one could identify specific clusters of cancer type, IVC and chemotherapy regimen in which unexpectedly beneficial outcomes or exceptional responses occur frequently enough to justify focused clinical trials.

## Supporting Information

S1 TREND Checklist TREND Checklist. (PDF)

S1 Protocol Clinical trial protocol. (DOC)

S1 Text Individual values for vitamin C and oxalic acid concentrations and individual plasma vitamin C time-course profiles. (PDF)

S2 Text Detailed case information. (PDF)
