# Supplementary material for: High-Dose Intravenous Vitamin C Combined with Cytotoxic Chemotherapy in Patients with Advanced Cancer: A Phase I-II Clinical Trial
Source: PLoS One. 2015 Apr 7;10(4):e0120228. doi: 10.1371/journal.pone.0120228 (PMC4388666; doi:10.1371/journal.pone.0120228)
Supplement: S1 Protocol — (DOC) [file pone.0120228.s002.doc]

Protocol S1 Clinical trial protocol

As of October 21, 2009, protocol unchanged with annual continuing review and approval by Jewish General Hospital Research Ethics Committee through 2013 when closed.

**Phase I - II clinical trial of conventional cytotoxic chemotherapy supplemented with intravenous vitamin C in patients with advanced cancer or hematologic malignancy for whom cytotoxic chemotherapy alone is only marginally effective**

LJ Hoffer, P Kavan, D Melnychuk, WH Miller, Lady Davis Institute for Medical Research, McGill University, and the Departments of Medicine and Oncology, Jewish General Hospital, Montreal

TABLE OF CONTENTS

1. SUMMARY AND INTRODUCTION

2. BACKGROUND AND RATIONALE

2.1 Vitamin C as a cancer treatment

2.2 Ascorbic acid biochemistry, physiology, and toxicity

2.3 Preclinical and early clinical data regarding anti-cancer effects

2.4 Do antioxidants like ascorbic acid reduce the efficacy of chemotherapy?

2.5 Mechanism of action

2.6 Previous experience by the co-investigators

2.7 Potential adverse effects and toxicity in this trial

3. STUDY OBJECTIVES

4. STUDY DESIGN

4.1 Overview of study design

4.2 Patient inclusion criteria

4.3 Patient exclusion criteria

4.4 Overview of Efficacy and Pharmacological Measurements

5. ASCORBIC ACID PHARMACOLOY AND TOXICITY

6. PROTOCOL DETAILS

6.1 Rationale of trial Design

6.2 Study procedures

6.3 Pharmacokinetic study

6.4 Dose escalation and modification

6.5 Ascorbic acid and cytotoxic drug dose modifications

7. ASSESSMENTS

7.1 Treatment efficacy and pharmacologic and metabolic measurements

7.2 Adverse event reporting

8. DATA ANALYSIS

8.1 Efficacy analysis

8.2 Pharmacologic and metabolic analyses, including pharmacokinetic studies

8.3 Quality of life outcomes analysis

8.4 Safety and side-effect analysis

9. DATA HANDLING, CONFIDENTIALITY AND ETHICS

10. CONSENT FORMS

11. REFERENCES

12. APPENDICES

**1. SUMMARY AND INTRODUCTION**

**Name of protocol:** Phase I - II clinical trial of conventional cytotoxic chemotherapy supplemented with intravenous vitamin C in patients with advanced cancer or hematologic malignancy for whom cytotoxic chemotherapy alone is only marginally effective

**Indication:** Patients with a variety of advanced cancers or hematologic malignancies for whom cytotoxic chemotherapy is indicated but, on average, only minimally to marginally effective.

**Phase I-II concept as applied to patients being offered cytotoxic chemotherapy for poorly responsive cancers:** Cytotoxic chemotherapy is relatively ineffective for a large proportion of common cancers. Some cytotoxic drugs are classified as effective even though they temporarily induce stable disease or an objective response in only a small proportion of patients. Even the modest achievement of doubling a typical response rate of 25% to 50% would benefit cancer patients enormously.

This clinical trial represents the first step in a systematic exploration of the hypothesis that supplementing certain chemotherapy regimens with therapy with a redox-active molecule like vitamin C (ascorbic acid; AA) could increase their anti-cancer activity or protect host tissues from toxicity with no loss of anti-cancer activity. The paradigm of supplementing chemotherapy with redox therapy has theoretical, basic and clinical support; it has reached the stage where careful and rigorous empirical clinical trials are appropriate. Both the American and Canadian Cancer Societies specifically advocate such research. However, until now, clinical trials of combination chemotherapy-redox therapy have been small, poorly designed, and unsystematic. Appropriate examination of this treatment paradigm requires a systematic, empirical approach similar to the one used in conventional cytotoxic drug discovery. Data suggesting a benefit of redox therapy are strongest for vitamin C. This protocol describes a Phase I clinical trial of promising chemotherapy-vitamin C combinations to determine their acceptability, limiting toxicity, and clinical benefit in patients with relentlessly progressive cancers for which conventional chemotherapy is clinically indicated but known to be only minimally or marginally effective. Good responses to chemotherapy being unusual, cancer-chemotherapy-vitamin combinations associated with a good response will be flagged and priority given to this cancer type and chemotherapy regimen as new participants volunteer for enrollment. Patients who previously received chemotherapy and patients currently receiving chemotherapy that has proven to be ineffective will be eligible to participate either by continuing the current chemotherapy with the addition of intravenous vitamin C or by starting a different chemotherapy regimen supplemented with intravenous vitamin C.

**Anti-cancer effects of vitamin C:** In millimolar concentrations, vitamin C in the form of ascorbic acid (AA) is cytotoxic to many cancer cells but innocuous to normal ones. Cancer-cytotoxic concentrations can be achieved in experimental animals and humans after parenteral injection. Vitamin C has anti-cancer efficacy in rodent cancer models. Several case reports lend plausibility to the notion that intravenous AA (IVAA) could have a role in treating some cancers. We completed a Phase I trial of IVAA in 24 patients with a variety of incurable cancers and haematological malignancies; 6 patients received the maximum tested dose of 1500 mg/kg body weight infused over 90 to 120 minutes three times per week. No evidence of toxicity emerged.

AA catalyses formation of hydrogen peroxide in cell culture media and in the extracellular fluid of experimental animals. Extracellular hydrogen peroxide diffuses into cells where it is converted to the hydroxyl free radical, which is selectively toxic to many cancer cells. Since most cytotoxic anti-cancer drugs generate reactive oxygen species, it is rational to predict that AA could augment the anti-cancer effect of standard chemotherapy. On the other hand, under most conditions AA is an antioxidant, raising the possibility that AA might reduce the efficacy of anti-cancer chemotherapy. Notwithstanding this concern, systematic reviews of randomized clinical trials indicate that the combination of chemotherapy with antioxidant therapy is either neutral or increases treatment response or survival with no effect on chemotherapy-related adverse effects or a reduction in their frequency or severity.

**Summary of study objectives:** The supplementation of cytotoxic chemotherapy with IVAA could prove to be an effective treatment for some patients with advanced malignancies. The primary objectives of this study are to identify a tolerable and safe dose of IVAA as a supplement to cytotoxic chemotherapy, to monitor for preliminary evidence that supplementing certain specific chemotherapy regimens with IVAA reduces the frequency or severity of serious adverse reactions to them (possibly increasing the number of chemotherapy cycles patients can tolerate), and to monitor for preliminary evidence that IVAA supplementation increases the efficacy of chemotherapy in specific cancers. Secondary objectives are to monitor biochemical and metabolic correlates of chemotherapy plus IVAA administration, the effect of chemotherapy on AA pharmacokinetics, and to monitor for improvements in mood and quality of life that may accrue from this treatment approach.

**Summary of study design:** This is a Phase I-II dose escalation study to evaluate the clinical safety, tolerance to and efficacy of IVAA given as a supplement to standard chemotherapy in the first-, second- or third-line treatment of advanced human cancers or hematologic malignancies. Approximately 24 patients will be enrolled.

**Test (and pharmacokinetic study) dose *versus* therapeutic doses:** During the week before on-protocol chemotherapy starts participants will be administered a test dose of IAA: 600 mg/kg body weight administered by continuous infusion over 90 minutes in the context of a pharmacokinetic study. The same test dose of 600 mg/kg over 90 minutes will be administered a second time during the first or second week of chemotherapy in order to determine the effect of chemotherapy on AA metabolism. There will be two therapeutic doses of IVAA; 900 mg/kg and 1500 mg/kg. The largest of these doses requires approximately 100 to 120 minutes for administration. The first 3 study participants will receive the 900 mg/kg therapeutic dose and if it is tolerated, the therapeutic dose will increase to 1500 mg/kg for subsequent cycles. The therapeutic dose for later participants will be 1500 mg/kg starting immediately in Cycle 1.

**2. BACKGROUND AND RATIONALE**

**2.1 Vitamin C as a cancer treatment:** In the early 1970's a Scottish cancer surgeon, Ewan Cameron, and a physician, Allan Campbell, administered intravenous and oral vitamin C to 50 consecutive hospitalized patients with advanced, untreatable cancer and carefully recorded the results. Important objective cancer remissions occurred in approximately one-fifth of the patients (1,2). Subsequently, two controlled clinical trials of treatment with 10 g/day AA taken orally, the first in patients with a variety of terminal cancers that had failed all previous therapy, and the second in patients with untreatable colorectal cancer but a good performance status, were carried out at the Mayo Clinic in Minnesota. When both trials turned out negative (3,4), mainstream oncologists dismissed the notion that vitamin C could play a role in cancer treatment. However, an important difference between the two negative Mayo Clinic studies and the highly promising Scottish studies (1,2,5,6) was never addressed, namely, that in the Scottish studies vitamin C was administered intravenously for the first 10 days of therapy and occasionally thereafter, whereas vitamin C was administered only orally in the Mayo Clinic trials. Subsequent research has shown that even extremely large oral doses of vitamin C do not increase circulating AA concentrations > 250 µmol/L, whereas intravenous administration raises concentrations up to 100 times higher; it is only the latter concentrations that are cytotoxic to some cancer cells. This new evidence has led to a call by mainstream researchers for a re-examination of the role of AA in cancer therapy

(7-15).

IVAA is widely and legally used as a cancer treatment by alternative practitioners (16-20) but regrettably, the details of therapy and its outcomes are seldom reported. An exception was H.D. Riordan, a physician who pioneered the use of IVAA in his clinic in Wichita, Kansas. Riordan and his colleagues published detailed information about their protocol, the resulting serum AA concentrations, and the clinical responses of some of their patients (16,21-23). As used in Riordan’s clinic, IVAA provides peak plasma AA concentrations > 20 mmol/L and sustained concentrations > 10 mmol/L for many hours. Infusions are given 2 to 3 times per week during a typical treatment program. According to Riordan, no toxicity and few if any adverse effects occurred among thousands of patients administered this treatment, and he published case reports of responses attributable to it (16,19,21,22,24), some of them independently verified as authentic at the U.S. National Cancer Institute when analysed according to NCI guidelines for identifying novel cancer therapies (20). Two patients with advanced ovarian cancer whose standard first-line chemotherapy was supplemented by 60 g IVAA twice weekly; there was no toxicity attributable to the AA infusions and indications that toxicity due to standard chemotherapy may have been reduced. Both patients had complete remissions (25).

**2.2 Ascorbic acid biochemistry, physiology, and toxicity:** AA is a water-soluble vitamin (vitamin C) similar in structure to glucose. Dietary AA is essential for life in primates, guinea pigs and certain fruit-eating birds and bats, all of which have lost or inactivated the gene for the key enzyme necessary to synthesize AA from glucose (26). Prolonged vitamin C deficiency leads to the fatal disease, scurvy. AA is a moderately strong reducing agent, and its known biological functions are related to its maintenance of the normal activity of iron-metalloenzymes by keeping the iron atom in the reduced state. Additionally, AA is an outstanding in vivo antioxidant (27) with measurable beneficial biologic effects (26,28,29).

Many Canadian and American people routinely consume megadoses of vitamin C. There is no evidence that even extremely large doses are toxic, carcinogenic, teratogenic, or cause adverse reproductive effects (26). AA has been administered intravenously in doses as high as 200 g/day without adverse consequence (30). Because of its limited absorption from the gastroinestinal tract, large oral doses act as an osmotic laxative. AA is broken down to oxalic acid, and it has frequently been claimed that high-dose AA causes oxalate kidney stones. Reports of AA-induced urolithiasis are difficult to evaluate because oxalate stones have a high natural prevalence. A formal epidemiologic study of healthy individuals failed to find any association between high-dose vitamin C consumption and kidney stone formation (26). Some data indicate that systemic conditioning develops by which plasma AA concentrations drop following discontinuation of a previously high intake of the vitamin (31,32). A recent expert review concluded that the evidence is inconsistent and does not suggest that systemic conditioning occurs to any significant extent (26).

Under certain conditions AA is metabolized to the pro-oxidant ascorbyl free radical. Because free reduced iron catalyses free radical formation, concerns have been expressed about the administration of high dose AA to patients with systemic iron overload (30,33-35). Notwithstanding these concerns, IVAA is used clinically to mobilize iron stores and improve anemia in erythropoietin-resistant hemodialysis patients (36). Large oral doses of AA are not pro-oxidant (26,29,37). In vitro studies indicate that under some conditions AA induces lipid hydroperoxide decomposition to DNA-damaging (“clastogenic”) intermediates, even in the absence of mineral catalysis (38); evidence further supporting this concept was recently published, but only in concentrations which are not attained when the vitamin is taken by mouth but are possible when it is administered intravenously (39). Thus, in some situations vitamin C appears to act an antioxidant and in others a pro-oxidant (39,40). When administered intravenously in large doses AA is at least partially a pro-oxidant, and this pro-oxidant activity mediates anti-cancer effects.

There are two reports of intravascular hemolysis in people infused with more than 50 g AA and one that describes cases of hemolysis in persons who received at least 6 g of AA as a single oral dose. All of the cases were in persons with the rare congenital disease, *glucose-6-phosphate dehydrogenase* deficiency, in which red blood cells lack the ability to reduce hydrogen peroxide because of deficient synthesis of reduced glutathione, the substrate for *glutathione peroxidase* (41).

Vitamin C is metabolized to oxalate, which is excreted in the urine. Serum oxalate concentrations are elevated in end-stage renal failure. Thus, serum oxalate concentrations were 17 µmol/L in normal persons but 75 µmol/L in patients with end-stage renal failure (42). Hyperoxalemia has occurred in persons with end-stage renal disease administered repeated large doses of AA (41), and oxalate nephropathy has occurred in patients with pre-existing renal insufficiency who were administered massive doses of IVAA (43,44). In our recent Phase I trial, which enrolled patients with normal renal function, the maximum IVAA dose of 1500 mg/kg was associated with the excretion of approximately 80 mg of oxalate over the period of the infusion and the subsequent six hours, a physiologically inconsequential amount (45).

A Phase I clinical trial of IVAA monotherapy was carried out in 24 patients with end-stage metastatic gastrointestinal cancers (46). IVAA was given by continuous infusion in doses ranging from 0.15 to 0.71 g/kg per day for up to 8 weeks. Side effects were minor and consisted largely of nausea, dry skin, dry mouth, fatigue, and edema, all of which are common in late stage advanced cancer. Standard biochemistry and hematology profiles were unaffected. One patient with a prior history of kidney stone developed a kidney stone after 13 days of treatment, possibly attributable to the IVAA. One patient experienced stable disease and continued treatment for 4 years (46). Because this protocol tested a continuous infusion of small doses of AA, average plasma AA concentrations in this study were only ~ 1.1 mmol/L and did not exceed 3.8 mmol/L. When large doses of AA are infused over 60 to 90 minutes the peak plasma concentration is predictably much higher, as illustrated by the following calculation. If a dose of 60 g of AA (303 mmol) distributes in the extracellular fluid volume (~ 14 L), the resulting peak plasma AA concentration should be > 20 mmol/L. In agreement with this logic, we found that the peak AA concentration after IVAA can be accurately predicted using the simple empirical formula: plasma AA concentration (g/L) = 3.75 D/W, where D is the ascorbic acid dose in g and W is the patient’s body weight in kg (47).

We recently completed a Phase I trial of IVAA administered 3 times per week to 24 patients with a variety of advanced malignancies there was no toxicity at any dose and the adverse effects encountered were mild and attributable to the effects of the rapid infusion of any hypertonic solution with resulting thirst and osmotic diuresis. These mild effects could be ameliorated or prevented by an adequate oral fluid intake during the infusions (47).

Several clinical trials of intravenous vitamin C as cancer therapy are at present underway in the United States (see Table 1).

**Table 1. Clinical Trials of Intravenous Vitamin C as Cancer Therapy Registered in the United States***

| Identification No. | Title | Phase |
| --- | --- | --- |
| NCT00441207 | Study of High-Dose Intravenous (IV) Vitamin C Treatment in Patients With Solid Tumors | I |
| NCT00803530 | Study of Association of Arsenic Trioxide (ATO) and Ascorbic Acid in Myelodysplastic Syndromes | II |
| NCT00671697 | Decitabine, Arsenic Trioxide and Ascorbic Acid for Myelodysplastic Syndromes and Acute Myeloid Leukemia | I |
| NCT00691210 | A Study to Evaluate the Use of the Drug Combination Vorinostat, Niacinamide, and Etoposide in Patients With Lymphoid Malignancies That Have Reoccurred | I |
| NCT00590603 | Trisenox, Ascorbic Acid and Bortezomib in Patients With Relapsed/Refractory Multiple Myeloma | I-II |
| NCT00621023 | Cephalon Decitabine, Arsenic Trioxide and Ascorbic Acid for Myelodysplastic Syndrome | II |
| NCT00803530 | Study of Association of Arsenic Trioxide (ATO) and Ascorbic Acid in Myelodysplastic Syndromes | II |
| NCT00954525 | Intravenous Vitamin C in Combination With Standard Chemotherapy for Pancreatic Cancer** | I |
| NCT00626444 | Pilot Trial of Intravenous Vitamin C in Refractory Non-Hodgkin Lymphoma (NHL) | I |
|  |  |  |

*http://www.cancer.gov/clinicaltrials, accessed September 16, 20009

**Patients will receive 50, 75 or 100 g IV ascorbic acid three times per week for 8 weeks plus gemcitabine and erlotinib; Kimmel Cancer Center, Thomas Jefferson University, Philadelphia

**2.3 Preclinical and early clinical data regarding anti-cancer effects of ascorbic acid:**

In 1969 Benade et al (48) reported an anti-cancer effect of AA in Ehrlich ascites carcinoma cells. Many other laboratories have confirmed and extended this observation in a large number of cancer cell types (49-70). Co-incubation of AA with cytoxic drugs increases their anti-cancer activity or protects noncancerous cells from chemotherapy toxicity (71-80). AA has anti-cancer activity when administered as sole treatment in tumour-bearing experimental animals (81-84). Also in tumour-bearing experimental animals, AA supplementation of cytoxic therapy reduced its toxicity (85,86) or increased its anti-cancer activity (81,86,87). The anti-cancer activity of AA is increased by vitamin K (57,63,65,80,88-98).

**2.4 Do antioxidants like ascorbic acid reduce the efficacy of chemotherapy?** Most cytotoxic drugs generate oxygen free radicals that mediate or contribute to their action. It is conceivable that AA, acting as a pro-oxidant, could augment the anti-cancer effect of standard chemotherapy. However, AA is also an antioxidant, and oncologists have been concerned that antioxidants like AA might reduce the efficacy of anti-cancer chemotherapy (99). Many reviews have addressed the question of whether concurrent antioxidant and chemotherapy improves or worsens the clinical outcome (65,100-112). The consensus view is that supplementation of chemotherapy with antioxidants is plausible and promising in some situations, but unproven. Historically, the American and Canadian Cancer Societies advised people with cancer to avoid antioxidants during or shortly after cytotoxic chemotherapy. In view of the emerging evidence, both organizations are no longer advise against the use antioxidants during chemotherapy, and call for clinical research to determine the potential benefits and risks of this approach.

**2.5 Mechanism of action:** It has long been suggested that the cancer-specific cytotoxicity of AA is at least largely mediated by extracellular hydrogen peroxide formation (48,49). A large amount of subsequent evidence supports this view (13,14,39,50,55,56,62,68,71,77,83,90-92,95,96). In high concentrations AA generates the ascorbyl free radical, which catalyses hydrogen peroxide formation both in cell culture systems and within the extracellular fluid of living experimental animals in a dose-responsive manner (13,62,83,92).

Cell culture studies indicate that AA and other antioxidants have greater inhibitory or toxic effects on malignant cells than normal ones (49); the mechanism is uncertain (113) and may relate to the faster growth rate of malignant cells (54). Some malignant cells may be deficient in the free radical protective enzyme, *catalase*, rendering them susceptible to the damaging effects of hydrogen peroxide, or deficient in *superoxide dismutase* (114).

Malignant cells accumulate AA (115), but AA does not need to enter cancer cells to exert an anti-cancer effect since it induces hydrogen peroxide formation in the extracellular space. It is of interest that AA is concentrated by cancer cells to much higher concentrations than in normal cells or the normal tissues surrounding cancer deposits (67,116-121). High intracellular AA concentrations increase the cytotoxicity of hydrogen peroxide, presumably by mobilizing intracellular reduced iron, which catalyses intracytosolic hydroxyl radical formation via the Fenton mechanism (69,122). Since vitamin C depleted cells from scorbutic guinea pigs accumulate AA to much higher concentrations than non-depleted cells (123), the introduction of high-dose AA to cancer patients with pre-existing vitamin C deficiency could induce a potent anti-cancer effect (124). Hydrogen peroxide depletes intracellular ATP. Consequently, cells that largely depend on glycolysis to generate ATP -- the Warburg effect -- could be especially susceptible to hydrogen peroxide toxicity (13,98). Such tumours could be clinically identified using conventional PET scanning, which images the intracellular concentration of radioactive fluorodeoxyglucose. Concentrations of hypoxia inducible factor (HIF), a master transcription factor whose biological functions are highly pertinent to cancer biology and the Warburg effect, are regulated by ascorbate-dependent prolyl hydroxylases (125-127).

Studied in cultured cells, intracellular AA loading (which can be accomplished by incubating cancer cells with dehydroascorbic acid) increases their resistance to cytotoxic drugs even when incubation with AA increases their response to them (55,93,128).

**2.6 Previous experience by the co-investigators:** We completed a Phase I clinical trial of monotherapy with IVAA in 24 patients with a variety of incurable malignancies. There was no toxicity (45,47). This work was favourably cited in a lead editorial in the Proceedings of the National Academy of Sciences (14). A TPD-approved Phase I-II clinical trial of IVAA in combination with carboplatin/docetaxel in non-small cell lung cancer is currently underway in this institution (TPD control No. 125663 and file No. 9427-JO832-41C). One patient completed the protocol without complications.

**2.7 Potential adverse effects and toxicity in this trial:** The most serious potential danger with IVAA is acute tumour lysis. Acute haemorrhage occurred within a few days of commencing IVAA in a small number of patients with advanced, terminal cancer (1,129). In two cases an autopsy was performed and demonstrated acute hemorrhage and necrosis specific to the primary tumour and its metastatic deposits (1). It is possible these catastrophic events occurred because the patients were overtly vitamin C deficient and hence prone to a more dramatic treatment effect (124). According to Riordan, no such adverse effects have occurred in thousands of infusions given in his clinic in the United States (personal communication). A more likely important adverse effect is not due to AA, but the sodium infused along with it. A 60 g dose of AA contains 344 mmol of sodium, approximately the amount of sodium in 2 liters of normal (0.9%) saline. Patients with uncontrolled heart failure, borderline cardiac function, or severe sodium-retaining states could be at risk of overt volume overload. Contrary to expectation, our limited experience in patients with fluid overload is that IVAA has a diuretic effect. In our clinical protocol IVAA is administered in a solution with an osmolality of up to 900 mOsmol/L, and almost the entire dose is promptly excreted with a lower osmolality in urine. IVAA thus acts as an osmotic diuretic similar to conventional osmotic diuretics like mannitol. Hyperglycemia can interfere with AA transport into cells (130). This is not a side effect of AA, but suggests that the benefit of AA therapy might be reduced in the presence of hyperglycemia. High serum AA concentrations are known to interfere with a number of common clinical biochemistry tests and by inhibiting *glucose oxidase* can falsely indicate low blood glucose (131).

Our recently completed phase I clinical trial of IVAA enrolled only patients with a serum creatinine concentration < 175 µmol/L (47). Because of the possibility of adverse effects from oxalic acid accumulation in patients with subnormal renal function, only patients whose serum creatinine concentration is no greater than 175 µmol/L will be enrolled, and the treatment will be temporarily or completely stopped if serum creatinine concentrations exceed this level during the course of the trial.

**3. STUDY OBJECTIVES**

**Primary Objectives**

1. To determine the safety and tolerability of intravenous ascorbic acid (IVAA) administered in a target dose of 1500 mg/kg as a supplement to cytotoxic chemotherapeutic drug therapy.

2. To obtain preliminary indications as to whether the addition of IVAA to cytotoxic chemotherapy reduces the frequency or severity of chemotherapeutic side effects and toxicity, potentially allowing patients to complete more chemotherapy cycles, to their benefit.

3. To monitor for anti-cancer efficacy when IVAA is given together with chemotheapy and then continues for a further 16 weeks after the final chemotherapy cycle; further extension may be considered in selected cases.

**Secondary Objectives**

1. To monitor biochemical and metabolic correlates of IVAA administration in the setting of chemotherapy.

2. To determine IVAA pharmacokinetics and urinary excretion of AA and it metabolites in patients treated with chemotherapy.

3. To monitor for preliminary improvements in quality of life and mood.

**4. STUDY DESIGN**

**4.1 Conceptual overview of study design and duration:** This is a Phase I-II dose escalation study of IVAA supplementation of best available chemotherapy to treat cancers for which this chemotherapy is associated with only a minimal to marginal objective clinical response. The addition of IVAA in close relation to standard cytotoxic drugs therapy could augment the anti-cancer effect of some cytotoxic drugs in patients who would fail to respond when chemotherapy or AA are given separately. IVAA may mitigate the toxicity of chemotherapy, allowing more patients to complete more cycles of effective chemotherapy, to their benefit (107).

The participating clinical oncologists will administer conventional chemotherapy in the Segal Cancer Centre of the Jewish General Hospital according to the principles of modern evidence-based medicine and current standard of care guidelines. IVAA will be administered in the Clinical Investigation Unit. The days on which IVAA is administered will be selected to balance the competing objectives of (1) maximum exposure to AA and (2) the need for a treatment protocol that is practical and efficient for patients and medical personnel. The selected regimen involves AA infusions on days that bracket chemotherapy infusion days, with AA infusions 2 or 3 times per week for as long as chemotherapy is clinically indicated. To allow for the possibility of an anti-tumour effect of maintenance IVAA alone in patients who responded to combined chemotherapy-IVAA, patients who responded to chemotherapy-IVAA will be offered up to 16 weeks of twice-weekly IVAA after the termination of the chemotherapy. If patients continue to be in remission after 16 weeks of twice-weekly IVAA, once weekly infusions will continue for an additional 12 months or longer.

Although the results of our recent trial of IVAA monotherapy, and those from an ongoing Phase I clinical trial which combines IVAA and carboplatin/docetaxel in non-small cell lung cancer do not suggest any increase in chemotherapy-related toxicity or side effects, this possibility will be formally examined for, and it is the stated primary objective of the study. The IVAA dose will be escalated using 2 doses already shown to be without side effects in our previous Phase I study of IVAA alone. These doses are 900 mg/kg and 1500 mg/kg IVAA per infusion.

The IVAA infusion protocol to be used in this study was developed in light of pharmacokinetic knowledge about IVAA and its metabolism. Hydrogen peroxide appears very quickly after a sufficiently high AA concentration is achieved either in cell culture media or in the extracellular fluid of a rat (13,39). We know from our previous Phase I trial of IVAA alone that 1500 mg/kg AA rapidly increases plasma AA to > 20 mmol/L by the end of the infusion, and remains > 10 mmol/L for more than 4 hours in people with normal renal function. In experimental animals, higher plasma (and extracellular fluid) AA concentrations are associated with proportionally greater extracellular fluid ascorbate radical and hydrogen peroxide concentrations up to the highest AA concentration achieved in the rat, 8 mmol/L (13). From these results we judge it appropriate to test the highest safe and tolerated dose of AA used in our previous Phase I trial (47), namely 1500 mg/kg, and to continue using our existing technique of infusing the dose at a maximum rate of approximately 1 g/h.

Patients considered for participation will have good performance status but a relentlessly advancing cancer or hematological malignancy for which current best therapy offers only a minimal to marginal likelihood of inducing a clinically meaningful response. Potential participants include patients offered first-line chemotherapy for non-small cell lung cancer, cancer of the esophagus, stomach, duodenum, gallbladder and pancreas, primary hepatocellular cancer, metastatic adenocarcinoma of unknown origin, advanced prostate cancer, metastatic melanoma, metastatic urothelial cancer, metastatic endometrial cancer, and most sarcomas. Cancers for which current standard of care second-line chemotherapy is known on average to be only marginally beneficial include the above cancers as well as cancer of the small intestine, colon and rectum. Although enrollment will not be restricted to any particular malignancies, the focus will be on common chemotherapy-resistant cancers such as cancer of the pancreas, stomach, or colon. Chemotherapeutic regimens of particular interest will include the antimetabolite drugs such as gemcitabine and capecitabine, because of experimental data suggesting an interaction (132) and suggestive clinical experience (Hoffer and Melnychuk, unpublished observations). Patients who received or are currently receiving chemotherapy but whose disease has progressed in spite of it will be eligible to continue receiving either the same chemotherapy or other chemotherapy without interruption when they are enrolled; indeed, such patients will be a focus of interest. It noteworthy that a Phase I clinical trial supplementing IVAA with chemotherapy in metastatic cancer of the pancreas is currently underway at Jefferson University, Pennsylvania (see Table 1).

**4.2. Patient inclusion criteria:**

1. Histologically or cytologically documented advanced or metastatic cancer or hematologic maligancy.

2. Physical functional status equivalent to Eastern Cooperative Oncology Group (ECOG) performance status 0 to 1 (Appendix A).

3. Age equal to or greater than 18 years.

4. Measurable disease as defined by at least one lesion that can be accurately measured in at least one dimension of 2 cm or greater by conventional techniques or 1 cm or greater by spiral CT scan as measured within 28 days prior to the planned start date of study treatment, in accordance with Response Evaluation Criteria in Solid Tumors (RECIST) criteria (see Appendix D). Note that patients with radiation to the only site of measurable disease will deem the patient ineligible unless definite progression is documented at the site after radiation treatment is complete.

5. Adequate bone marrow, hepatic, renal and cardiac function documented within 14 days prior to enrollment, and suitability for the clinically indicated conventional chemotherapy according to the normal standard of care.

6. No current calcium oxalate nephrolithiasis with the potential to reduce urinary flow.

7. Feasible to come to the clinical research unit 2 to 3 times weekly for intravenous infusions.

8. Life expectancy of at least 8 weeks.

9. Signed informed consent.

10. Patients must be accessible for complete documentation of the treatment, toxicity, response assessment and follow-up at the Clinical Research Unit of the Jewish General Hospital.

11. Patients with asymptomatic or controlled brain metastases will be eligible and may receive dexamethasone as clinical indicated; however, they must have completed radiotherapy or radiosurgery at least 1 week prior to enrollment.

12. Patients who are receiving anti-cancer therapy but whose disease progresses despite it may enter the protocol and continue the same therapy or start a different chemotherapy regimen without interruption.

**4.3. Patient exclusion criteria:**

1. Any cancer for which existing conventional treatment offers more than a 33% likelihood of an clinically meaningful response.

2. Serum creatinine > 175 µmol/L.

3. Serious gastrointestinal disorders including active bleeding.

4. Patients with serious or uncontrolled infections, cardiac or neurological conditions.

5. Major surgery within 4 weeks of study drug administration or local radiation therapy within 7 days.

6. Dementia or severely altered mental status that would render informed consent impossible.

7. Women of childbearing potential must use an acceptable form of contraception for the duration of the study, and must have a pregnancy test within 7 days of the first administration of chemotherapy.

8. Lactating women.

9. *Glucose-6-phosphate dehydrogenase* deficiency.

10. Any abnormal laboratory value or medical condition that would, in the investigators’ judgement, make the patient a poor candidate for study.

**4.4. Overview of efficacy and pharmacological measurements:**

1. Tumour Response, stable disease (no progression), and progression.

2. Side effect of profile in comparison to what historically occurs with standard chemotherapy.

3. Biomarkers (plasma and monocyte AA concentration, serum C-reactive protein).

4. Quality of life and mood questionnaire (FACT-G; POMS).

5. Pharmacokinetic analysis prior to chemotherapy and 3 days after chemotherapy in the first month of treatment only.

**5. DESCRIPTION OF ASCORBIC ACID**

**5.1 Ascorbic acid:** Ascorbic acid is a 6-carbon ketolactone structurally related to glucose and other hexoses. Its formal chemical name is 3-oxo-L-gulofuranolactone, C_6_H_8_O6̧ molecular weight 176. Ascorbic Acid Injection USP is provided by Alveda Pharma, a the sole Canadian distributor of this product, which is manufactured by the Canadian pharmaceutical company, Bioniche Pharma. The Canadian Drug Identification Number (DIN) is 02245214. The product is a clear, colourless to slightly yellow sterile solution of AA in water for injection, for intravenous, intramuscular, or subcutaneous use. Each mL contains AA 500 mg (2.84 mmol), edetate disodium 0.025%, and water for injection q.s. with the pH (range 5.5 to 7.0) adjusted with sodium bicarbonate, hence providing ~ 2.84 mmol sodium. The product is supplied in sterile 50 mL single use glass bottles containing 500 mg/mL of ascorbic acid USP. The stock solution contains 2.84 mmol of AA per mL and 2.84 mmol of sodium per mL for a theoretical osmolality of 2.84 + 2.84 = 5.7 mOsm/mL.

The product is stored in a carton protected from light between 2 and 8 degrees C. It must not be frozen. IVAA is to be infused within 4 hours of preparing diluted solutions for injection. When the dose is less than 15 g the stock solution is diluted with lactated Ringer’s to guarantee sufficiently high osmolality to permit intravenous administration. When the dose is equal to or greater than 15 g, the infusate is brought to the required concentration using sterile water to achieve an osmolality between 500 and 900 mOsm/L. The osmolality maybe calculated from the molecular weight of AA assuming it is completely dissociated in aqueous solution. Examples are given as follows.

15 g (30 mL stock qs 300 mL using sterile water): 570 mOsm/L.

30 g (60 mL stock qs 500 mL using water): 684 mOsm/L.

60 g (120 mL stock qs 900 mL using water): 760 mOsml/L.

70 g (140 mL stock qs 1100 mL using water): 725 mOsml/L.

80 g (160 mL stock qs 1100 mL using water): 829 mOsml/L.

90 g (180 mL stock qs 110 mL using water): 933 mOsml/L.

100 g (200 mL stock qs 1200 mL using water): 950 mOsml/L.

110 g (220 mL stock qs 1400 mL using water): 896 mOsml/L.

120 g (240 mL stock qs 1400 mL using water): 977 mOsml/L.

130 g (260 mL stock qs 1600 mL using water): 926 mOsml/L.

140 g (280 mL stock qs 1800 mL using water): 887 mOsml/L.

Intravenous infusions are administered via a peripheral or central intravenous catheter at a rate of 0.5 g/minute to approximately 1 g/minute, and not usually faster than approximately 1 g/minute. At the dose of 1.5 g/kg an infusion will typically last 105 minutes.

Potential side effects of IVAA (described in detail earlier) include oxalate renal stones, oxalate nephropathy in patients with pre-existing renal insufficiency, intravascular hemolysis in patients with *glucose-6-phosphate dehydrogenase* deficiency, fluid overload due to the high sodium content of the intravenous ascorbic acid/ascorbate preparation, thirst and a diuretic effect due to the hyperosmolality of the infusate with excretion of > 90% of the dose of sodium ascorbate over the subsequent 6 hours. Apparent tumour haemorrhage occurred in a small number of patients who received IVAA in a case series published in 1974 (1), but has not subsequently been described despite administration to thousands of patients in the United States. The risk may therefore be considered very slight or zero. We observed no evidence of oxalate stone in our recently-completed Phase I trial of IVAA alone, nor any evidence of more than a physiologically transient trivial increase in urinary oxalate excretion on the day of an IVAA infusion (45).

**6. PROTOCOL DETAILS**

**6.1 Rationale of trial design:** The study concept is to augment the effects or mimimize toxicity of chemotherapy by administering IVAA in close proximity to chemotherapy administration. When chemotherapy is given by weekly intravenous infusion (as with conventional gemcitabine monotherapy on days 1, 8, and 15 of each 28 day cycle) AA will be infused 2 or 3 days per week on non-chemotherapy days and on two days during the “rest” week when chemotherapy is not administered. When chemotherapy is administered intravenously at 3 week intervals (as with combination carboplatin and gemcitabine or a taxane), AA will be infused one day prior to and 2 more times during the first week of the cycle, and twice weekly during the second and third week. Patients who receive weekly intravenous chemotherapy will receive IVAA either 2 or 3 days of each chemotherapy week and 2 times on the “weeks off.” Patients administered oral chemotherapy (as with capecitabine) will have AA infusions 2 or 3 times per week for as long as chemotherapy continues. If tumour progression occurs after a minimum of 2 months of therapy the protocol will terminate. As in standard clinical practice, patients whose treatment results in stable disease or an objective response may continue receiving IVAA and chemotherapy at the discretion of the treating oncologist. Some patients may respond to chemotherapy-IVAA but have to discontinue chemotherapy because of unacceptable toxicity. In these cases IVAA will be offered twice weekly for a further 16 weeks after the final chemotherapy cycle with continuing evaluation of tumour status every 8 weeks (or earlier if clinically indicated). Patients who remain in remission for 16 weeks after the final chemotherapy will be considered for indefinite once weekly IVAA. Patients whose disease progresses will not continue to receive IVAA.

**6.2 Study procedures**: For patients who continue on protocol (i.e., stable disease or tumour response) the entire treatment protocol could last for several months. Disease progression will trigger discontinuation of the IVAA. The trial schema is shown in the following tables. Details of the scheme will vary slightly depending on the nature of the chemotherapy protocol selected by the treating oncologist.

**Table 2. First month of protocol**

| Measurement or Action | Screening Period | Day -7 to -1 | Day 1  (M) | Day 2  (T) | Day 3  (W) | Day 5  (F) | Days 8-12 | Days 15-28 |
| --- | --- | --- | --- | --- | --- | --- | --- | --- |
| Medical history, informed consent, and screening tests | X [1] |  |  |  |  |  |  |  |
| ECOG status | X |  |  |  |  |  |  |  |
| Vital signs | X |  | X [2] | X [2] |  |  |  |  |
| Physical exam | X |  | X [2] |  |  |  |  |  |
| Safety labs | X |  | X [3] | X [3] |  |  |  |  |
| Biomarkers [4] | X |  | X |  |  |  |  |  |
| CT scan [5] | X |  |  |  |  |  |  |  |
| FACT -G and POMS questionnaires |  |  | X |  |  |  |  |  |
| Commence chemotherapy |  |  |  | X |  |  |  |  |
| IVAA 600 mg/kg with pharmacokinetic sampling |  | X |  |  |  | X[6] |  |  |
| IVAA administration |  |  | X |  | X | X[7] | X [7] | X [7] |

[1] One-time only screening tests: pregnancy test, red blood cell *G6PD* activity, and ECG. Hematology, coagulation, biochemistry and urinalysis are measured repeatedly; they must be obtained within 2 weeks prior to the first chemotherapy dose in Cycle 1. A test dose of IVAA (with pharmacokinetics) is administered prior to Cycle 1 only and the same dose on day 3.

[2] Physical exam and ECOG can be done up to one week before dosing.

[3] For day one of Cycle 1, the repeat blood measurements (“safety labs”) are done only if screening tests were performed more than 2 weeks prior to Day 1.

[4] Biomarkers include serum C-reactive protein (main biochemistry laboratory) and plasma and white blood cell AA (Dr. Hoffer’s laboratory), as well as common tumour cell markers specific to the individual cancer (e.g. CEA for colon cancer, PSA for prostate cancer).

[5] Baseline CT scan is normally carried out within 4 weeks of protocol Day 1. Thereafter a CT scan is carried out at 2 month intervals.

[6] Only in the first month. The IVAA dose on protocol day 5 is 600 mg/kg over 90 minutes with blood and urine sampling for pharmacokinetic analysis.

[7] IVAA is administered two or three times per week on days that are at least one day apart (e.g. Mon-Wed, Mon-Thur, Mon-Fri, Tue-Thur, Tue-Fri, Wed-Fri).

As chemotherapy and IVAA continue, ECOG status, vital signs, physical exam, safety labs and biomarker levels appropriate for the cancer type will be obtained or recorded from the oncology clinic record for protocol purposes at the beginning of every treatment month. A CT scan will be obtained towards the end of every second month. During chemotherapy all patients are normally evaluated weekly by their oncologist, and this commonly includes standard safety labs. The oncology clinic is in close proximity to the CRU. Patients can therefore easily come to the CRU for study purposes.

**Explanatory remarks:** The minimum required baseline screening, study procedures, and end-of-study evaluations are indicated in the table above. Other parameters and/or increased frequency of examinations or clinical follow-up may be needed for patient management depending on findings emerging from the study. In cases when chemotherapy has to be discontinued because of toxicity the protocol will continue with no chemotherapy and IVAA given twice weekly as long as there is stable disease (absence of disease progression) for 16 weeks after the end of the last chemotherapy cycle, reassessed at periods of 2 months. In the event of scheduling conflicts, study evaluations will take place prior to dosing on the designated Day 1 ± 3 days, or as specified in the following sections.

In addition to the procedures listed in the tables, additional blood samples will be taken to monitor for hematological toxicities according to the standard of patient care.

**Screening:** The evaluation of disease status will be completed within 4 weeks prior to dosing. Hematology and coagulation, chemistry, and urinalysis must be done within 2 weeks prior to dosing. As part of screening, all patients will undergo a complete medical history, including concomitant treatments, full clinical examination (including vital signs, ECOG performance status, baseline signs and symptoms with adverse event assessments using CTCAE version 3.0 grading, height and body weight), a 12-lead resting electrocardiogram, and a CT scan. The following laboratory tests will be obtained.

Hematology (CBC, differential, PT/INR, PTT, and erythrocyte G6PD screen. (The G6PD screen is done only once and may be carried out any time prior to entry).

Biochemistry (serum creatinine, urea, sodium, potassium, chloride, bicarbonate, magnesium, calcium, phosphorus, glucose, total bilirubin, ALT, ALP, GGT, LDH, total protein, uric acid, albumin, and calcium).

Routine urinalysis.

Serum b-HCG (premenopausal women of childbearing potential).

Serum C-reactive protein.

Plasma and monocyte AA concentrations.

**End of study assessments:** If disease progression occurs the patient the study is completed and IVAA is discontinued.

**Follow-up visit:** Study drug related adverse events, including laboratory test results ≥ grade 2, will be followed until resolution, return to baseline grade, or deemed irreversible unless the patient withdraws consent or receives additional treatment for his/her disease. Patients will be monitored at least once a month. Adverse events may be considered irreversible if no improvement is observed in two consecutive visits. Patients who withdraw from the study due to DLTs will be monitored until all toxicity has been resolved or returns to baseline unless the patient withdraws consent to follow up or receives another treatment for his/her disease.

**Subject withdrawal:** Patients may choose to withdraw from the trial at any time or they may be withdrawn at any time at the discretion of the investigator or sponsor for safety, behavioural, or administrative reasons. If a patient does not return for a scheduled visit, every effort should be made to contact the patient. In any circumstance, every effort should be made to document subject outcome, if possible. If the subject withdraws consent, no further evaluations should be performed and no attempts should be made to collect additional data.

**6.3 Pharmacokinetic study:** There will be two formal pharmacokinetic studies to plasma and urinary AA metabolism in response to a standard 600 mg/kg dose of AA infused over 90 minutes at baseline and on the third day after Cycle 1 chemotherapy. The measurements will consist of a blood sample and urine sample collection prior to IVAA, one sample drawn 60 minutes after the start of the IVAA infusion, and an “end of infusion” (EOI) sample at the 90 minute time point, and sequential samples at time points one, two, three and four hours after the end of infusion. There will be concurrent timed complete urine collections to measure AA and oxalic acid, a metabolite of ascorbic acid catabolism.

The blood sample is drawn from an antecubital vein or central venous catheter distant from the infusion catheter. A 1 mL sample is drawn to clear the sampling catheter and discarded. The next 1.0 mL was drawn into a chilled heparinized test tube, immediately centrifuged at 5 degrees C for 10 minutes and 0.5 ml of the resulting plasma mixed immediately with 2.0 mL of ice cold 90% methanol 10% 1 mmol/L EDTA and analyzed immediately or frozen at - 80 degrees C until analysis. The analysis is by HPLC with electrochemical detection using an ESA Coulochem III detector equipped with a 5011A analytical cell (133). With this detection methodology the chromatographic peak area is highly linear up to a concentration of 10 mmol/L. For concentrations in this range and higher, the sample is diluted 10-fold in EDTA/methanol prior to injection on the column.

These analyses are carried out to verify that the IVAA increases plasma AA concentrations under baseline conditions as found in our earlier Phase I trial and to determine AA metabolism in the setting of concurrent chemotherapy. This is an important determination because it has previously been observed that the plasma concentration of AA is markedly reduced and its metabolic clearance increased in critical illness (134,135), including after high-dose interleukin-2 administration to cancer patients (136). It will be important to document to what extent the rise in plasma AA concentration and its urinary excretion are blunted by the inflammatory state induced by the chemotherapy.

Occasional samples will be drawn to measure plasma AA concentrations in selected patients at the discretion of the investigators as clinically indicated. Biomarkers include are serum C-reactive protein, and plasma and white blood cell AA concentrations.

**6.4 IVAA dose escalation and modification:** Only two doses of IVAA will be studied. The lower dose of 900 mg/kg will be used for the first 3 participants, and only in the first month of the protocol. If no dose-limiting toxicity (DLT) attributable to IVAA occurs during this month, the IVAA dose will increase to 1500 mg/kg. If No DLT attributable to IVAA occurs for these 3 participants, subsequent participants will receive 1500 mg/kg IVAA starting immediately. Patients are likely to experience some toxicity from their chemotherapy. The toxicity profiles of these drugs are known and recognizable. The responses to make when these toxicities occur will be in line with ordinary clinical practice guidelines. What would define DLT due to IVAA? When considered in relation to IVAA a dose limiting toxicity (DLT) will be defined as any of the following:

1. Acute pulmonary edema not attributed to chemotherapy.

2. Oxalate renal colic or urinary tract obstruction.

3. Heinz-body positive intravascular hemolysis.

The Maximum Administered Dose (MAD) of IVAA is the dose at which 2 or more out of 3 to 6 patients experience one of the above DLTs during Cycle 1. The Maximum Tolerated Dose (MTD) will be defined as the highest dose level below the MAD at which none or one out of 6 patients experience a Cycle 1 DLT. If no DLTs occur and there is no more than one grade 2 adverse event (CTCAE, as defined by the NCI Common Terminology Criteria for Adverse Events v3.0 (CTCAEAE). http://ctep.info.nih.gov/reporting/CTCAE.html) among the first 3 patients during their first cycle, the dose of IVAA will be increased to 1500 mg/kg for subsequent participants. If there is no DLT but two or more grade 2 CTCAE occur in the first cycle, the dose escalation will change to the more conservative dose of 1200 mg/kg for the first cycle in the subsequent 3 participants, and increase again to 1500 mg/kg if they are found to tolerate 1200 mg/kg infusions in the first cycle.

IVAA is administered in a hypertonic concentration (750 to 950 mosmol/L). The infusion will temporarily increase plasma osmolarity, causing an osmotic diuresis and thirst in all people and light-headedness in some, as occurs during the infusion of all osmotic diuretics such as mannitol. These minor side effects can be mitigated or prevented by asking patients to drink water from the beginning of the infusion, and, if necessary, by moderately slowing the IVAA infusion rate. These side effects are not to be considered toxic reactions to IVAA.

**IVAA dose modification if IVAA causes clinically important adverse effects at any time in any cycle:** If, in the judgement of the physician, it is certain or very likely that a patient is having clinically important adverse effects that are caused by the IVAA rather than the chemotherapy, and the dose is at 1500 mg/kg per infusion, the dose will be reduced to 1200 mg/kg for the remainder of that cycle, and then increased to 1500 mg/kg for the subsequent cycle. If the IVAA-attributable adverse effect occurs in the final cycle, the dose will be increased to 1500 mg/kg for the twice weekly infusions that continue for up to 16 weeks in the absence of chemotherapy. If the dose of 1500 mg/kg is again judged to be inducing clinically important adverse effects, the dose will once again be reduced to 1200 mg/kg per infusion.

**IVAA dose modification if the renal function deteriorates importantly for any reason:** The recommended Phase II dose of IVAA was determined in patients with serum creatinine < 175 µmol/L. Because the risk of oxalate nephropathy is unknown for patients with poor renal function, IVAA will be withheld if the serum creatinine concentration exceeds 175 µmol/L

and only resumed once the concentration has fallen below this level.

**6.5 IVAA and chemotherapy dose modification**: Patients experiencing IVAA-related DLTs may be continue treatment with a reduced IVAA dose. Chemotherapy dose modifications will made on the basis of the highest CTCAE grade of treatment-related toxicity during the previous cycle. Dose modifications may occur either as a dose reduction or a dose delay pending adequate recovery, thus extending the length of the previous cycle.

**Dose modifications and interruption of IVAA:** Patients developing a DLT, toxicity, or serious adverse effect that is either plainly or strongly inferentially attributable to IVAA may have their IVAA interrupted for one or several doses and continue to receive only chemotherapy. Upon adequate recovery, treatment with IVAA may be resumed at the same or lower dose. Interruption of IVAA for more than 1 cycle will result in discontinuation of the patient from the study. Patients experiencing chemotherapy toxicity will continue receiving IVAA unless, in the judgment of physicians, there is a strongly plausible reason to believe that the IVAA is contributing to and worsening the adverse effect. Upon adequate recovery, IVAA treatment will resume at the same IVAA dose level. Patients who experience intolerable chemotherapy toxicity but have completed at least 1 month of therapy and had a sustained response or stable disease will continue treatment with twice-weekly IVAA alone for up to 16 weeks; if disease progression has occurred after the final chemotherapy administration or subsequently during the 16 week period of IVAA alone, the protocol will stop.

**Other medications:** Commercially available palliative and supportive care for disease-related symptoms will be offered. Patients may use various nutritional supplements at their discretion, in consultation with the investigators, as long as this use is carefully documented. Megestrol acetate, pamidronate or other supportive care agents for palliation of disease-related symptoms are allowed. Chronic systemic corticosteroid is permitted. Low dose steroid use for the control of nausea and vomiting is allowed, including prophylactic use. Treatment-related adverse events will be treated in accordance with clinical care. In keeping with standard clinical practice, colony stimulating factors may be administered prophylactically during the study period in cases of recurrent febrile neutropenia, recurrent afebrile neutropenia with a suspected underlying infection, recurrent afebrile grade 3-4 neutropenia in the setting of objective response or severe bone marrow suppression complicated by a suspected infection.

**7. ASSESSMENTS**

The following assessments will be made:

1. Tumour response, occurrence of progression, and time to progression.

2. Side effect of profile in comparison to what historically occurs with standard chemotherapy in comparable patients.

3. Biomarkers (plasma and circulating monocyte AA concentration, C-reactive protein as well as cancer-specific biomarkers in clinical use).

4. Quality of life and mood questionnaire (FACT-G and POMS).

**7.1 Treatment efficacy and pharmacologic and metabolic measurements:** All patients who have received a minimum of 2 cycles of study treatment will be considered as evaluable for response. Disease and response assessments are defined by the Response Evaluation Criteria in Solid Tumors (RECIST; see Appendix D). Additional evaluations of measurable and non-measurable target lesions may be scheduled, as appropriate, using the same method of assessment (physical examination, X-ray, CT scan or MRI) throughout the study. Changes in tumour size will be categorized as complete response (CR), partial response (PR), stable disease (SD), or progressive disease (PD), the latter incorporating the appearance of new lesions. For patients with either complete or partial response, additional assessments will be required for confirmation of response. Imaging studies will be repeated at least every two months, normally 7-10 days prior to the start of the next dose and a final time upon discontinuation from the study, unless the imaging study has been performed within the last 4 weeks.

Patients who develop new brain metastases while on study may have treatment interrupted to receive a course of cranial radiation and restart study treatment after a recovery period of one week. In cases where radiologic assessment of the brain was not performed during screening, a new baseline evaluation will be made prior to restarting treatment.

**Biomarkers and pharmacokinetics:** There will be two formal pharmacokinetic studies to measure AA pharmacokinetics at baseline and on the third day after Cycle 1 chemotherapy. The dose used in these pharmacokinetic studies is 600 mg/kg administered at a constant rate over 90 minutes. The measurements will consist of a blood sample and urine sample collection prior to IVAA, a sample 60 minutes into the into the IVAA infusion, and an “end of infusion” (EOI) sample at the 90 minute time point, with samples taken at time points one, two, three and four hours after the end of infusion. There will be concurrent timed complete urine collections. Additionally, occasional samples will be drawn to measure plasma AA concentrations in selected patients at the discretion of the investigators as clinically indicated. Biomarkers include are serum C-reactive protein (hospital biochemistry lab) and plasma and white blood cell AA (Dr. Hoffer’s laboratory), as well as cancer-specific biomarkers used clinically (e.g., CEA in colon cancer and PSA in prostate cancer).

**Quality of life and mood assessments:** Quality of life and symptoms will be measured using two questionnaires. The first is the FACT-G questionnaire, a widely used, validated questionnaire used in cancer patients that measures four domains of quality of life (physical well-being, functional well-being, social/family well-being, emotional well-being; see http://www.facit.org/). The second questionnaire is the extensively validated Profile of Mood States^TM^ Brief form (POMS-Brief). Both questionnaires are available in French and English. The questionnaire will be completed by the patient with the assistance of the research nurse. (Appendix D).

**7.2 Adverse event reporting:** All observed or volunteered adverse events will be recorded. Information will be obtained to determine the outcome of the adverse event and determine its cause. Follow-up of the adverse event, after the date of therapy discontinuation, is required if the adverse event or its sequelae persist. Follow-up is required until the event or its sequelae resolve or stabilize at a level acceptable to the investigator.

**Definition of an adverse event:** An adverse event is any untoward medical occurrence in a clinical investigation subject administered a product or medical device; the event need not necessarily have a causal relationship with the treatment or usage. Examples of adverse events include but are not limited to abnormal test findings, clinically significant symptoms and signs, changes in physical examination findings, hypersensitivity, progression/worsening of underlying disease, and lack of effect. Additionally, they may include the signs or symptoms resulting from drug overdose, drug withdrawal, drug abuse, drug misuse, drug interactions, drug dependency, extravasation, or exposure *in utero*. Progression of the malignancy under study is not an adverse event. However, if the malignancy has a fatal outcome during the trial, disease progression must be recorded as an adverse event (and an SAE with CTC grade 5).

**Abnormal laboratory findings:** The criteria for determining whether an abnormal objective test finding should be reported as an adverse event are as follows. The test result is associated with accompanying symptoms, and/or requires additional diagnostic testing or medical/surgical intervention, and/or leads to a change in trial dosing or discontinuation from the study, significant additional concomitant drug treatment or other therapy, and/or is considered to be an adverse event by the investigator or sponsor.

Merely repeating an abnormal test, in the absence of any of the above conditions, does not constitute an adverse event. Any abnormal test results that is determined to be an error does not require reporting as an adverse event

**Serious adverse events:** A serious adverse event or serious adverse drug reaction is any untoward medical occurrence at any dose that either results in death, is life-threatening, requires inpatient hospitalization or prolongation of existing hospitalization, results in a persistent or significant disability/incapacity, or results in congenital anomaly/birth defect. Medical and scientific judgment should be exercised in determining whether an event is an important medical event. An important medical event may not be immediately life-threatening and/or result in death or hospitalization. However, if it is determined that the event may jeopardize the subject and may require intervention to prevent one of the other outcomes listed in the definition above, the important medical event should be reported as serious.

**Discontinuations:** The reason for a subject discontinuing from the trial will be recorded in the CRF. A discontinuation occurs when an enrolled subject ceases participation in the study, regardless of the circumstances, prior to completion of the protocol. The investigator must determine the primary reason for discontinuation.

**8. DATA ANALYSIS**

**8.1. Efficacy analysis:**  The efficacy of chemotherapy plus IVAA will be assessed by objective tumour response and time to disease progression. Tumour response is assessed as explained in Appendix D. Objective response rate and stable disease rate are defined as the percent of evaluable study participants who demonstrate an objective response (CR and PR) and stable disease. The duration of a CR will be measured from the date the CR was first recorded to the date of disease progression is documented (taking as the reference for progression the lowest measured tumour size recorded from the time the treatment started). The duration of overall response will be calculated for all responding patients (CR and PR). It will be defined as the time elapsed from the first recorded CR or PR until documentation of progression (taking as the reference for progression the lowest measured tumour size level recorded since the treatment started). The time to disease progression is defined as the time from the date of enrollment to the date of documented disease progression or death from any cause. If a patient has neither progressed nor died, his or her data will be censored on the date of the last disease assessment.

**8.2 Pharmacologic and metabolic analyses, including pharmacokinetic studies:** An important aim of the study is to determine the effect of chemotherapy on AA pharmacokinetics. The acute-phase response induced by chemotherapy can greatly increase the metabolic clearance of AA (135). A pharmacokinetic analysis will be carried out in each participant prior to starting chemotherapy and on the third day after chemotherapy in Cycle 1. On each occasion IVAA will be infused in a dose of 600 mg/kg over 90 minutes. Plasma AA will be drawn prior to the infusion, 60 and 90 minutes into the infusion, and 1, 2, 3 and 4 hours after the end of the infusion, with a complete urine collection. A standard pharmacokinetic analysis of metabolic clearance and distribution volume will be carried out as described in (135). A small number of additional samples will be taken to measure plasma AA in patients from time to time during the study as clinically indicated.

**8.3 Quality of life outcomes analysis:** The FACT-G and POMS questionnaire results will provide the opportunity to obtain exploratory information about the effects of the treatment

**8.4 Safety and side-effect analysis:** Safety data will be summarized by dose levels for all treated patients using appropriate tabulations and descriptive statistics. All patients who receive at least one dose of study medication will be considered evaluable for safety analyses. The analysis of safety will extend through 30 days after the last administration of study drug. Safety analyses will include tabulation of the following:

1. Worst CTCAE grade adverse events (1) in the first cycle and (2) during the study.

2. Worst CTCAE grade lab abnormalities (1) in the first cycle and (2) during the study.

**9. DATA HANDLING, CONFIDENTIALITY AND ETHICS**

Prior to implementation the study protocol will have been approved by Health Canada and by the Research Ethics Committee of the Jewish General Hospital. The trial will be performed in accordance with good clinical practise and clinical research guidelines. Chemotherapy will be administered in accordance with the best current standard of care as used in the Clinical Research Unit. No samples for genetic or DNA analysis will be obtained in this study. Case information will be maintained in the Clinical Research Unit as is standard practise. The sponsor of the study is within the CRU, so no information will leave the CRU.

**10. CONSENT FORMS**

Printed separately.

**11. REFERENCES**

1. Cameron E, Campbell A. The orthomolecular treatment of cancer. II. Clinical trial of high-dose ascorbic acid supplements in advanced human cancer. Chem Biol Interact 1974;9:285-315.

2. Cameron E, Campbell A, Jack T. The orthomolecular treatment of cancer. III. Reticulum cell sarcoma: double complete regression induced by high-dose ascorbic acid therapy. Chem Biol Interact 1975;11:387-93.

3. Creagan ET, Moertel CG, O'Fallon JR et al. Failure of high-dose vitamin C (ascorbic acid) therapy to benefit patients with advanced cancer. N Engl J Med 1979;301:687-90.

4. Moertel CG, Fleming TR, Creagan ET, Rubin J, O'Connell MJ, Ames MM. High-dose vitamin C versus placebo in the treatment of patients with advanced cancer who have had no prior chemotherapy. N Engl J Med 1985;312:137-41.

5. Campbell A, Jack T, Cameron E. Reticulum cell sarcoma: two complete 'spontaneous' regressions, in response to high-dose ascorbic acid therapy. A report on subsequent progress. Oncology 1991;48:495-7.

6. Cameron E, Campbell A. Innovation vs. quality control: an 'unpublishable' clinical trial of supplemental ascorbate in incurable cancer. Medical Hypotheses 1991;36:185-9.

7. Block G, Henson DE, Levine M. Vitamin C: a new look. Ann Intern Med 1991;114:909-10.

8. Henson DE, Block G, Levine M. Ascorbic acid: biologic functions and relation to cancer. J Natl Cancer Inst 1991;83:547-50.

9. Padayatty SJ, Levine M. Reevaluation of ascorbate in cancer treatment: emerging evidence, open minds and serendipity. J Am Coll Nutr 2000;19:423-5.

10. Batist G. Clinical evaluation of vitamin C and other micronutrients. J Orthomolecular Med 2000;15:189-92.

11. Hoffer LJ. Proof versus plausibility: rules of engagement for the struggle to evaluate alternative cancer therapies. CMAJ 2001;164:351-3.

12. Tamayo C, Richardson MA. Vitamin C as a cancer treatment: state of the science and recommendations for research. Altern Ther Health Med 2003;9:94-101.

13. Chen Q, Espey MG, Sun AY et al. Ascorbate in pharmacologic concentrations selectively generates ascorbate radical and hydrogen peroxide in extracellular fluid in vivo. Proc Natl Acad Sci USA 2007;104:8749-54.

14. Frei B, Lawson S. Vitamin C and cancer revisited. Proc Natl Acad Sci USA 2008;105:11037-8.

15. Ohno S, Ohno Y, Suzuki N, Soma G, Inoue M. High-dose vitamin C (ascorbic acid) therapy in the treatment of patients with advanced cancer. [Review] [56 refs]. Anticancer Res 2009;29:809-15.

16. Riordan NH, Riordan HD, Meng X, Li Y, Jackson JA. Intravenous ascorbate as a tumor cytotoxic chemotherapeutic agent. Medical Hypotheses 1995;44:207-13.

17. Gaby AR. Intravenous nutrient therapy: the "Myer's Cocktail". Altern Med Rev 2002;7:389-403.

18. Riordan HD, Hunninghake RB, Riordan NH et al. Intravenous ascorbic acid: protocol for its application and use. P R Health Sci J 2003;22:287-90.

19. Gonzalez MJ, Miranda-Massari JR, Mora EM et al. Orthomolecular oncology review: ascorbic acid and cancer 25 years later. Integ Cancer Ther 2005;4:32-44.

20. Padayatty SJ, Riordan HD, Hewitt SM, Katz A, Hoffer LJ, Levine M. Intravenously administered vitamin C as cancer therapy: three cases. CMAJ 2006;174:937-42.

21. Riordan HD, Jackson JA, Schultz M. Case study: high-dose intravenous vitamin C in the treatment of a patient with adenocarcinoma of the kidney. J Orthomolecular Med 1990;5:5-7.

22. Riordan NH, Jackson JA, Riordan HD. Intravenous vitamin C in a terminal cancer patient. J Orthomolecular Med 1996;11:80-2.

23. Riordan NH, Riordan HD, Casciari JJ. Clinical and experimental experiences with intravenous vitamin C. J Orthomolecular Med 2000;15:201-13.

24. Riordan HD, Riordan NH, Jackson JA et al. Intravenous vitamin C as a chemotherapy agent: a report on clinical cases. P R Health Sci J 2004;23:115-8.

25. Drisko JA, Chapman J, Hunter VJ. The use of antioxidants with first-line chemotherapy in two cases of ovarian cancer. J Am Coll Nutr 2003;22:118-23.

26. Panel on Dietary Antioxidants and Related Compounds, Subcommittees on Upper Reference Levels of Nutrients and Interpretation and Uses of Dietary Reference Intakes, Standing Committee on the Scientific Evaluation of Dietary Reference Intakes. Dietary reference intakes: vitamin C, vitamin E, selenium, and beta-carotene and other carotenoids. Washington, DC: National Academy Press, 2000.

27. Frei B, England L, Ames BN. Ascorbate is an outstanding antioxidant in human blood plasma. Proc Natl Acad Sci USA 1989;86:6377-81.

28. Fraga CG, Motchnik PA, Shigenaga MK, Helbock HJ, Jacob RA, Ames BN. Ascorbic acid protects against endogenous oxidative DNA damage in human sperm. Proc Natl Acad Sci USA 1991;88:11003-6.

29. Polidori MC, Mecocci P, Levine M, Frei B. Short-term and long-term vitamin C supplementation in humans dose-dependently increases the resistance of plasma to ex vivo lipid peroxidation. Arch Biochem Biophys 2004;423:109-15.

30. Rivers JM. Safety of high-level vitamin C ingestion. Ann N Y Acad Sci 1987;498:445-54.

31. Tsao CS, Salimi SL. Evidence of rebound effect with ascorbic acid. Medical Hypotheses 1984;13:303-10.

32. Tsao CS, Leung PY. Urinary ascorbic acid levels following the withdrawal of large doses of ascorbic acid in guinea pigs. J Nutr 1988;118:895-900.

33. Young IS, Trouton TG, Torney JJ, McMaster D, Callender ME, Trimble ER. Antioxidant status and lipid peroxidation in hereditary haemochromatosis. Free Rad Biol Med 1994;16:393-7.

34. Chen WT, Lin YF, Yu FC, Kao WY, Huang WH, Yan HC. Effect of ascorbic acid administration in hemodialysis patients on in vitro oxidative stress parameters: influence of serum ferritin levels. Am J Kidney Dis 2003;42:158-66.

35. Chen K, Suh J, Carr AC, Morrow JD, Zeind J, Frei B. Vitamin C suppresses oxidative lipid damage in vivo, even in the presence of iron overload. Am J Physiol 2000;279:E1406-E1412.

36. Melendez O. Intravenous vitamin C for erythropoietin resistance. Semin Dialysis 2000;13:335-6.

37. Carr A, Frei B. Does vitamin C act as a pro-oxidant under physiological conditions? FASEB J 1999;13:1007-24.

38. Lee SH, Oe T, Blair IA. Vitamin C-induced decomposition of lipid hydroperoxides to endogenous genotoxins. Science 2001;292:2083-6.

39. Duarte TL, Almeida GM, Jones GD. Investigation of the role of extracellular H2O2 and transition metal ions in the genotoxic action of ascorbic acid in cell culture models. Toxicol Lett 2007;170:57-65.

40. Duarte TL, Lunec J. Review: When is an antioxidant not an antioxidant? A review of novel actions and reactions of vitamin C. Free Radic Res 2005;39:671-86.

41. Levine M, Rumsey SC, Daruwala R, Park JB, Wang Y. Criteria and recommendations for vitamin C intake. JAMA 1999;281:1415-23.

42. Balcke P, Schmidt P, Zazgornik J, Kopsa H, Deutsch E. Secondary oxalosis in chronic renal insufficiency. N Engl J Med 1980;303:944.

43. McAllister CJ, Scowden EB, Dewberry FL, Richman A. Renal failure secondary to massive infusion of vitamin C. JAMA 1984;252:1684.

44. Wong K, Thomson C, Bailey RR, McDiarmid S, Gardner J. Acute oxalate nephropathy after a massive intravenous dose of vitamin C. Aust N Z J Med 1994;24:410-1.

45. Robitaille L, Mamer OA, Miller WH, Jr. et al. Oxalic acid excretion after intravenous ascorbic acid administration. Metabolism 2009;58:263-9.

46. Riordan HD, Casciari JJ, Gonzalez MJ et al. A pilot clinical study of continuous intravenous ascorbate in terminal cancer patients. P R Health Sci J 2005;24:269-76.

47. Hoffer LJ, Levine M, Assouline S et al. Phase I clinical trial of intravenous ascorbic acid in advanced malignancy. Ann Oncol 2008;19:1969-1974..

48. Benade L, Howard T, Burk D. Synergistic killing of Ehrlich ascites carcinoma cells by ascorbate and 3-amino-1,2,4,-triazole. Oncology 1969;23:33-43.

49. Bram S, Froussard P, Guichard M et al. Vitamin C preferential toxicity for malignant melanoma cells. Nature 1980;284:629-31.

50. Liotti FS, Bodo M, Menghini AR, Guerrieri P, Pezzetti F. Antagonism between catalase and ascorbic acid in control of normal and neoplastic cell multiplication. Cancer Lett 1986;33:99-106.

51. Helgestad J, Pettersen R, Storm-Mathisen I et al. Characterization of a new malignant human T-cell line (PFI-285) sensitive to ascorbic acid. Eur J Haematol 1990;44:9-17.

52. Park CH, Kimler BF. Growth modulation of human leukemic, preleukemic, and myeloma progenitor cells by L-ascorbic acid. Am J Clin Nutr 1991;54:1241S-6S.

53. Kao TL, Meyer WJ3, Post JF. Inhibitory effects of ascorbic acid on growth of leukemic and lymphoma cell lines. Cancer Lett 1993;70:101-6.

54. Leung PY, Miyashita K, Young M, Tsao CS. Cytotoxic effect of ascorbate and its derivatives on cultured malignant and nonmalignant cell lines. Anticancer Res 1993;13:475-80.

55. Baader SL, Bruchelt G, Trautner MC, Boschert H, Niethammer D. Uptake and cytotoxicity of ascorbic acid and dehydroascorbic acid in neuroblastoma (SK-N-SH) and neuroectodermal (SK-N-LO) cells. Anticancer Res 1994;14:221-7.

56. Sestili P, Brandi G, Brambilla L, Cattabeni F, Cantoni O. Hydrogen peroxide mediates the killing of U937 tumor cells elicited by pharmacologically attainable concentrations of ascorbic acid: cell death prevention by extracellular catalase or catalase from cocultured erythrocytes or fibroblasts. J Pharmacol Exp Ther 1996;277:1719-25.

57. Venugopal M, Jamison JM, Gilloteaux J et al. Synergistic antitumour activity of vitamins C and K3 against human prostate carcinoma cell lines. Cell Biol Int 1996;20:787-97.

58. Sakagami H, Satoh K, Kochi M. Comparative study of the antitumor action between sodium 5,6- benzylidene-L-ascorbate and sodium ascorbate. Anticancer Res 1997;17:4451-2.

59. Osmak M, Kovacek I, Ljubenkov I, Spaventi R, Eckert-Maksic M. Ascorbic acid and 6-deoxy-6-chloro-ascorbic acid: potential anticancer drugs. Neoplasma 1997;44:101-7.

60. Koh WS, Lee SJ, Lee H et al. Differential effects and transport kinetics of ascorbate derivatives in leukemic cell lines. Anticancer Res 1998;18:2487-93.

61. Roomi MW, House D, Eckert-Maksic M, Maksic ZB, Tsao CS. Growth suppression of malignant leukemia cell line in vitro by ascorbic acid (vitamin C) and its derivatives. Cancer Lett 1998;122:93-9.

62. Makino Y, Sakagami H, Takeda M. Induction of cell death by ascorbic acid derivatives in human renal carcinoma and glioblastoma cell lines. Anticancer Res 1999;19:3125-32.

63. Jamison JM, Gilloteaux J, Taper HS, Summers JL. Evaluation of the in vitro and in vivo antitumor activities of vitamin C and K-3 combinations against human prostate cancer. J Nutr 2001;131:158S-60S.

64. Casciari JJ, Riordan NH, Schmidt TL, Meng XL, Jackson JA, Riordan HD. Cytotoxicity of ascorbate, lipoic acid, and other antioxidants in hollow fibre in vitro tumours. Br J Cancer 2001;84:1544-50.

65. Calderon PB, Cadrobbi J, Marques C et al. Potential therapeutic application of the association of vitamins C and K3 in cancer treatment. Curr Med Chem 2002;9:2271-85.

66. Kang JS, Cho D, Kim YI et al. Sodium ascorbate (vitamin C) induces apoptosis in melanoma cells via the down-regulation of transferrin receptor dependent iron uptake. J Cell Physiol 2005;204:192-7.

67. Casciari JJ, Riordan HD, Miranda-Massari JR, Gonzalez MJ. Effects of high dose ascorbate administration on L-10 tumor growth in guinea pigs. P R Health Sci J 2005;24:145-50.

68. Chen Q, Espey MG, Krishna MC et al. Pharmacologic ascorbic acid concentrations selectively kill cancer cells: Action as a pro-drug to deliver hydrogen peroxide to tissues. Proc Natl Acad Sci USA 2005;102:13604-9.

69. Riviere J, Ravanat JL, Wagner JR. Ascorbate and H2O2 induced oxidative DNA damage in Jurkat cells. Free Rad Biol Med 2006;40:2071-9.

70. Carosio R, Zuccari G, Orienti I, Mangraviti S, Montaldo PG. Sodium ascorbate induces apoptosis in neuroblastoma cell lines by interfering with iron uptake. Mol Cancer 2007;6:55-66.

71. Bruchelt G, Schraufstatter IU, Niethammer D, Cochrane CG. Ascorbic acid enhances the effects of 6-hydroxydopamine and H2O2 on iron-dependent DNA strand breaks and related processes in the neuroblastoma cell line SK-N-SH. Cancer Res 1991;51:6066-72.

72. Sarna S, Bhola RK. Chemo-immunotherapeutical studies on Dalton's lymphoma in mice using cisplatin and ascorbic acid: synergistic antitumor effect in vivo and in vitro. Arch Immunol Ther Exp 1993;41:327-33.

73. Chiang CD, Song EJ, Yang VC, Chao CC. Ascorbic acid increases drug accumulation and reverses vincristine resistance of human non-small-cell lung-cancer cells. Biochem J 1994;301:759-64.

74. Song EJ, Yang VC, Chiang CD, Chao CC. Potentiation of growth inhibition due to vincristine by ascorbic acid in a resistant human non-small cell lung cancer cell line. Eur J Pharmacol 1995;292:119-25.

75. Kurbacher CM, Wagner U, Kolster B, Andreotti PE, Krebs D, Bruckner HW. Ascorbic acid (vitamin C) improves the antineoplastic activity of doxorubicin, cisplatin, and paclitaxel in human breast carcinoma cells in vitro. Cancer Lett 1996;103:183-9.

76. Grad JM, Bahlis NJ, Reis I, Oshiro MM, Dalton WS, Boise LH. Ascorbic acid enhances arsenic trioxide-induced cytotoxicity in multiple myeloma cells. Blood 2001;98:805-13.

77. Bahlis NJ, McCafferty-Grad J, Jordan-McMurry I et al. Feasibility and correlates of arsenic trioxide combined with ascorbic acid-mediated depletion of intracellular glutathione for the treatment of relapsed/refractory multiple myeloma. Clin Cancer Res 2002;8:3658-68.

78. Abdel-Latif MM, Raouf AA, Sabra K, Kelleher D, Reynolds JV. Vitamin C enhances chemosensitization of esophageal cancer cells in vitro. J Chemother 2005;17:539-49.

79. Gokhale P, Patel T, Morrison MJ, Vissers MC. The effect of intracellular ascorbate on the susceptibility of HL60 and Jurkat cells to chemotherapy agents. Apoptosis 2006;11:1737-46.

80. Kassouf W, Highshaw R, Nelkin GM, Dinney CP, Kamat AM. Vitamins C and K3 sensitize human urothelial tumors to gemcitabine. J Urol 2006;176:1642-7.

81. Meadows GG, Pierson HF, Abdallah RM. Ascorbate in the treatment of experimental transplanted melanoma. Am J Clin Nutr 1991;54:1284S-91S.

82. Tsao CS. Inhibiting effect of ascorbic acid on the growth of human mammary tumor xenografts. Am J Clin Nutr 1991;54:1274S-80S.

83. Chen Q, Espey MG, Sun AY et al. Pharmacologic doses of ascorbate act as a prooxidant and decrease growth of aggressive tumor xenografts in mice. Proc Natl Acad Sci USA 2008;105:11105-9.

84. Belin S, Kaya F, Duisit G, Giacometti S, Ciccolini J, Fontes M. Antiproliferative effect of ascorbic acid is associated with the inhibition of genes necessary to cell cycle progression. PLoS ONE 2009.4:e4409.

85. Fujita K, Shinpo K, Yamada K et al. Reduction of adriamycin toxicity by ascorbate in mice and guinea pigs. Cancer Res 1982;42:309-16.

86. Shimpo K, Nagatsu T, Yamada K et al. Ascorbic acid and adriamycin toxicity. Am J Clin Nutr 1991;54:Suppl-1301S.

87. Taper HS, Roberfroid M. Non-toxic sensitization of cancer chemotherapy by combined vitamin C and K3 pretreatment in a mouse tumor resistant to oncovin. Anticancer Res 1992;12:1651-4.

88. De Loecker W, Janssens J, Bonte J, Taper HS. Effects of sodium ascorbate (vitamin C) and 2-methyl-1,4- naphthoquinone (vitamin K3) treatment on human tumor cell growth in vitro. II. Synergism with combined chemotherapy action. Anticancer Res 1993;13:103-6.

89. Taper HS, Keyeux A, Roberfroid M. Potentiation of radiotherapy by nontoxic pretreatment with combined vitamins C and K3 in mice bearing solid transplantable tumor. Anticancer Res 1996;16:499-503.

90. Sakagami H, Satoh K. Modulating factors of radical intensity and cytotoxic activity of ascorbate. Anticancer Res 1997;17:3513-20.

91. Sakagami H, Hosaka M, Arakawa H et al. Role of hydrogen peroxide in antitumor activity induction by sodium 5,6-benzylidene-L-ascorbate. Anticancer Res 1998;18:2519-24.

92. Asano K, Satoh K, Hosaka M et al. Production of hydrogen peroxide in cancerous tissue by intravenous administration of sodium 5,6-benzylidene-L-ascorbate. Anticancer Res 1999;19:229-36.

93. Sakagami H, Satoh K, Hakeda Y, Kumegawa M. Apoptosis-inducing activity of vitamin C and vitamin K. Cellular & Molecular Biology 2000;46:129-43.

94. Zhang W, Negoro T, Satoh K et al. Synergistic cytotoxic action of vitamin C and vitamin K3. Anticancer Res 2001;21:3439-44.

95. Gonzalez MJ, Miranda-Massari JR, Mora EM et al. Orthomolecular oncology: a mechanistic view of intravenous ascorbate's chemotherapeutic activity. P R Health Sci J 2002;21:39-41.

96. Dalton WS. Targeting the mitochondria: an exciting new approach to myeloma therapy. Clin Cancer Res 2002;8:3643-5.

97. Verrax J, Stockis J, Tison A, Taper HS, Calderon PB. Oxidative stress by ascorbate/menadione association kills K562 human chronic myelogenous leukaemia cells and inhibits its tumour growth in nude mice. Biochem Pharmacol 2006;72:671-80.

98. Verrax J, Vanbever S, Stockis J, Taper H, Calderon PB. Role of glycolysis inhibition and poly(ADP-ribose) polymerase activation in necrotic-like cell death caused by ascorbate/menadione-induced oxidative stress in K562 human chronic myelogenous leukemic cells. Int J Cancer 2007;120:1192-7.

99. D'Andrea GM. Use of antioxidants during chemotherapy and radiotherapy should be avoided. CA Cancer J Clin 2005;55:319-21.

100. Lamson DW, Brignall MS. Antioxidants in cancer therapy; their actions and interactions with oncologic therapies. Altern Med Rev 1999;4:304-29.

101. Conklin KA. Dietary antioxidants during cancer chemotherapy: impact on chemotherapeutic effectiveness and development of side effects. Nutr Cancer 2000;37:1-18.

102. Lamson DW, Brignall MS. Antioxidants and cancer therapy II: quick reference guide. Altern Med Rev 2000;5:152-63.

103. Drisko JA, Chapman J, Hunter VJ. The use of antioxidant therapies during chemotherapy. Gynecol Oncol 2003;88:434-9.

104. Conklin KA. Cancer chemotherapy and antioxidants. J Nutr 2004;134:3201S-4S.

105. Doyle C, Kushi LH, Byers T et al. Nutrition and physical activity during and after cancer treatment: an American Cancer Society guide for informed choices. CA: a Cancer Journal for Clinicians 2006;56:323-53.

106. Moss RW. Should patients undergoing chemotherapy and radiotherapy be prescribed antioxidants? Integ Cancer Ther 2006;5:63-82.

107. Block KI, Koch AC, Mead MN, Tothy PK, Newman RA, Gyllenhaal C. Impact of antioxidant supplementation on chemotherapeutic efficacy: a systematic review of the evidence from randomized clinical trials. Cancer Treatment Rev 2007;33:407-18.

108. Fang J, Nakamura H, Iyer AK. Tumor-targeted induction of oxystress for cancer therapy. J Drug Target 2007;15:475-86.

109. Block KI, Koch AC, Mead MN, Tothy PK, Newman RA, Gyllenhaal C. Impact of antioxidant supplementation on chemotherapeutic toxicity: a systematic review of the evidence from randomized controlled trials. Int J Cancer 2008;123:1227-39.

110. Lawenda BD, Kelly KM, Ladas EJ, Sagar SM, Vickers A, Blumberg JB. Should supplemental antioxidant administration be avoided during chemotherapy and radiation therapy? J Natl Cancer Inst 2008;100:773-83.

111. Block KI. An antioxidant turnabout. Integ Cancer Ther 2008;7:59-61.

112. Block K, Koch A, Mead M, Newman RA, Gyllenhaal C. Re: Should supplemental antioxidant administration be avoided during chemotherapy and radiation therapy? J Natl Cancer Inst 2009;101:124-5.

113. Cole WC, Prasad KN. Contrasting effects of vitamins as modulators of apoptosis in cancer cells and normal cells: a review. Nutr Cancer 1997;29:97-103.

114. Oberley LW, Buettner GR. Role of superoxide dismutase in cancer: a review. Cancer Res 1979;39:1141-9.

115. Agus DB, Vera JC, Golde DW. Stromal cell oxidation: a mechanism by which tumors obtain vitamin C. Cancer Res 1999;59:4555-8.

116. Kakar SC, Wilson CWM. Ascorbic acid values in malignant disease. Proc Nutr Soc 1975;35:9A-10A.

117. Anthony HM, Schorah CJ. Severe hypovitaminosis C in lung-cancer patients: the utilization of vitamin C in surgical repair and lymphocyte-related host resistance. Br J Cancer 1982;46:354-67.

118. Farber CM, Kanengiser S, Stahl R, Liebes L, Silber R. A specific high-performance liquid chromatography assay for dehydroascorbic acid shows an increased content in CLL lymphocytes. Anal Biochem 1983;134:355-60.

119. Langemann H, Torhorst J, Kabiersch A, Krenger W, Honegger CG. Quantitative determination of water- and lipid-soluble antioxidants in neoplastic and non-neoplastic human breast tissue. Int J Cancer 1989;43:1169-73.

120. Piyathilake CJ, Bell WC, Johanning GL, Cornwell PE, Heimburger DC, Grizzle WE. The accumulation of ascorbic acid by squamous cell carcinomas of the lung and larynx is associated with global methylation of DNA. Cancer 2000;89:171-6.

121. May JM, Li L, Hayslett K, Qu ZC. Ascorbate transport and recycling by SH-SY5Y neuroblastoma cells: response to glutamate toxicity. Neurochem Res 2006;31:785-94.

122. Duarte TL, Jones GD. Vitamin C modulation of H2O2-induced damage and iron homeostasis in human cells. Free Rad Biol Med 2007;43:1165-75.

123. Chatterjee M, Saluja R, Kumar V et al. Ascorbate sustains neutrophil NOS expression, catalysis, and oxidative burst. Free Rad Biol Med 2008;45:1084-93.

124. Hoffer LJ. Nutrients as biologic response modifiers. In: Quillan P, Williams RM, eds. Adjuvant Nutrition in Cancer Treatment. Arlington Heights, IL: Cancer Treatment Research Foundation 1993:55-79.

125. Semenza GL. Hydroxylation of HIF-1: oxygen sensing at the molecular level. Physiology 2004;19:176-82.

126. Brahimi-Horn MC, Chiche J, Pouyssegur J. Hypoxia signalling controls metabolic demand. Curr Opin Cell Biol 2007;19:223-9.

127. Gao P, Zhang H, Dinavahi R et al. HIF-dependent antitumorigenic effect of antioxidants in vivo. Cancer Cell 2007;12:230-8.

128. Heaney ML, Gardner JR, Karasavvas N et al. Vitamin C antagonizes the cytotoxic effects of antineoplastic drugs. Cancer Res 2008;68:8031-8.

129. Campbell A, Jack T. Acute reactions to mega ascorbic acid therapy in malignant disease. Scot Med J 1979;24:151-3.

130. Price KD, Price CS, Reynolds RD. Hyperglycemia-induced ascorbic acid deficiency promotes endothelial dysfunction and the development of atherosclerosis. Atherosclerosis 2001;158:1-12.

131. Freemantle J, Freemantle MJ, Badrick T. Ascorbate interferences in common clinical assays performed on three analyzers. Clin Chem 1994;40:950-1.

132. Chinery R, Brockman JA, Peeler MO, Shyr Y, Beauchamp RD, Coffey RJ. Antioxidants enhance the cytotoxicity of chemotherapeutic agents in colorectal cancer: a p53-independent induction of p21WAF1/CIP1 via C/EBPbeta. Nature Med 1997;3:1233-41.

133. Levine M, Wang Y, Rumsey SC. Analysis of ascorbic acid and dehydroascorbic acid in biological samples. Methods Enzymol 1999;299:65-76.

134. Schorah CJ, Downing C, Piripitsi A et al. Total vitamin C, ascorbic acid, and dehydroascorbic acid concentrations in plasma of critically ill patients. Am J Clin Nutr 1996;63:760-5.

135. Rumelin A, Humbert T, Luhker O, Drescher A, Fauth U. Metabolic clearance of the antioxidant ascorbic acid in surgical patients. J Surg Res 2005;129:46-51.

136. Marcus SL, Petrylak DP, Dutcher JP et al. Hypovitaminosis C in patients treated with high-dose interleukin 2 and lymphokine-activated killer cells. Am J Clin Nutr 1991;54:1292S-7S.

**12. APPENDICES**

**A. ECOG**

**B. Response Evaluation Criteria in Solid Tumors**

**C. NCI Common Terminology Crieteria for Adverse Events**

**D. Quality of Life and Symptoms Endpoint Questionnaire and brief Profile of Mood States^TM^ Questionnaire**

**APPENDIX A: EASTERN COOPERATIVE ONCOLOGY GROUP (ECOG) PERFORMANCE STATUS SCALE** **(125)**

Grade 0: Fully active, able to carry on all pre-disease performance without restriction

Grade 1: Restricted in physically strenuous activity but ambulatory and able to carry out work of a light or sedentary nature, e.g., light house work, office work

Grade 2: Ambulatory and capable of all self care but unable to carry out any work activities. Up and about more than 50% of waking hours

Grade 3: Capable of only limited self care, confined to bed or chair more than 50% of waking hours

Grade 4: Completely disabled. Cannot carry on any self care. Totally confined to bed or chair

**APPENDIX B: Response Evaluation Criteria in Solid Tumors**

Criteria for evaluation of objective tumour response will be as described by Therasee P, et al, New Guidelines to evaluate the response to treatment in solid tumors, JNCI 2000; 92:205-16.

Measurements of all lesions should be recorded in metric units. At baseline, all lesions should be categorized and documented either as (1) measurable Lesions (lesions that can be accurately measured as 2.0 cm in at least 1 dimension with conventional techniques or 1.0 cm with spiral CT scan, (2) nonmeasurable lesions (all other lesions, including small lesions, the longest diameter being <2.0 cm with conventional techniques or <1.0 cm with spiral CT scan) or (3) truly nonmeasurable lesions. Nonmeasurable lesions are bone lesions, leptomeningeal involvement, malignant ascites, malignant pleural and pericardial effusions, inflammatory breast disease, cutaneous or pulmonary lymphangitis, and abdominal masses which are not confirmed and followed by imaging techniques. Previously irradiated lesions are nonmeasurable except in cases of documented progression of the lesion since the completion of radiation therapy.

**Baseline Evaluation**

Baseline measurements must be performed not more than 4 weeks prior to the initiation of treatment. Patients must have at least one measurable lesion at baseline to be included in the analysis of this endpoint. If the measurable disease is restricted to a solitary lesion, its neoplastic nature should be confirmed by cytology/histology.

**Documentation of “Target” and “Nontarget” Lesions**

Lesions used for subsequent analysis must be documented as “target” and “nontarget” at baseline. Baseline documentation of tumor sites includes imaging assessment of disease in the chest, abdomen, and pelvis. Baseline imaging study of the brain is not required unless there is a prior history of brain metastasis or the patient has clinical symptoms of brain metastasis.

**Techniques for Assessing Measurable Disease**

The same method of assessment used at baseline should be used to characterize each identified and reported lesion during follow-up. Imaging-based evaluation is preferred to evaluation by clinical (physical) examination when both methods have been used to assess the antitumor effect of a treatment. Accepted methods of tumour assessment include:

Clinical Examination: Lesions detected by clinical examination will only be considered measurable when they are superficial (e.g., skin nodules and palpable lymph nodes).

Documentation of skin lesions by color photography, including a ruler to estimate the size of the lesion, is recommended.

Chest X-ray (CXR): Lesions on CXR are acceptable as measurable lesions when they are clearly defined and surrounded by aerated lung. However, computerized tomography (CT) is preferable.

CT, magnetic resonance imaging (MRI): Conventional CT and MRI should be performed with contiguous cuts of 10 mm or less in slice thickness. Spiral CT should be performed using a 5 mm contiguous reconstruction algorithm. This applies to tumors of the chest, abdomen, and pelvis. Head and neck and extremities require specific protocols.

Ultrasound: Ultrasound should not be used to measure tumor lesions not clinically accessible when the primary endpoint is objective response. It is, however, a possible alternative to clinical measurements of superficial palpable nodes, subcutaneous lesions, and thyroid nodules.

**Target Lesions**

Up to 10 total lesions, a maximum of 5 lesions per organ, that are representative of all involved organs may be selected and recorded as target lesions at baseline. Target lesions should be selected on the basis of their size (lesions with the longest diameter) and their suitability for accurate repetitive measurements (either by imaging techniques or clinically). A sum of the longest diameter (LD) for *all target lesions* will be calculated and reported as the baseline sum LD. The baseline sum LD will be used as reference to further characterize the objective tumor response of the disease.

**Nontarget Lesions**

All other lesions (or sites of disease) should be identified as nontarget lesions and recorded as nontarget lesions at baseline. Measurement of nontarget lesions at baseline is not required and should be recorded as “present.” Each nontarget lesion should be documented as either present or absent in each subsequent evaluation.

**Objective Response Classifications**

Response will be assessed and reported according to the following Response Evaluation Criteria in Solid Tumors (RECIST):

Complete Response (CR): Disappearance of all target and nontarget lesions. CR must be confirmed by repeat assessments performed no less than 4 weeks after the criteria for response are first met to qualify as CR;

Partial Response (PR): At least a 30% decrease in the sum of the longest diameter (LD) of target lesions taking as reference the baseline sum LD. Nontarget lesions may persist provided there is no unequivocal progression in these lesions. PR must be confirmed by

repeat assessments performed no less than 4 weeks after the criteria for response are first met to qualify as PR;

Progressive Disease (PD): At least a 20% increase in the sum LD of the target lesions from the smallest sum LD recorded since the beginning of therapy or the appearance of one or more new lesions or unequivocal progression of existing nontarget lesions; and

Stable Disease (SD): Measurements demonstrating neither sufficient shrinkage to qualify for PR nor sufficient increase to qualify as PD during the first 6 weeks after the start of treatment taking as reference the smallest sum LD since the treatment started. During this time, nontarget lesions may persist provided there is no unequivocal progression in these lesions.

**Frequency of Tumor Measurements**

Tumour measurements should be completed 6 weeks after initiation of therapy, except for confirmation of CR or PR. Additional tumor measurements may be completed as needed.

**Evaluation of Best Overall Response**

This is the best response recorded since start of treatment until disease progression or recurrence (taking as reference for PD the smallest measurement since treatment started). For CR and PR the best response assignment will depend on the achievement of both the initial establishment and confirmation of CR or PR. Determination of best overall response is as follows.

| Target Lesions | Nontarget lesions | New lesions | Overall response |
| --- | --- | --- | --- |
| CR | CR | No | CR |
| CR | Non-Cr/Non-PD | No | PR |
| PR | Non-PD | No | PR |
| SD | Non-PD | No | SD |
| PD | Any | Yes or No | PD |
| Any | PD | Yes or No | PD |
| Any | Any | Yes | PD |

**APPENDIX C: NCI COMMON TERMINOLOGY CRITERIA FOR ADVERSE EVENTS VERSION 3 (CTCAE V 3.0)**

A copy of the CTCAE V 3.0 can be downloaded from the CTEP homepage at http://ctep.cancer.gov/reporting/ctc.html.

**APPENDIX D: QUALITY OF LIFE AND SYMPTOMS ENDPOINT QUESTIONNAIRE AND PROFILE OF MOOD STATES^TM^ QUESTIONNAIRE, BRIEF FORMAT**

The Functional assessment of Chronic Illness Therapy Quality of life FACT-G questionnaire and symptoms endpoint questionnaire is described at http://www.facit.org/.

The brief POMS checklist, provided by MHS Multi-Health Systems, Inc., is a standard extensively validated 30 item questionnaire used both in normal and mood-altered people. The one page check list can be completed in a 5 minutes or less. The respondent rates each of several mood states as either “not at all,” “a little,” “moderately,” “quite a bit,” or “extremely.” French and English versions are available. Sample terms are friendly, tense, angry, worn out, unhappy, clear-headed, lively, confused, sorry for things done, shaky, listless, peeved, considerate, sad, active, on edge, grouchy, blue, energetic, panicky, hopeless, relaxed, unworthy, spiteful, sympathetic, uneasy, restless, unable to concentrate, fatigued, helpful, annoyed, discouraged, resentful, nervous, lonely, miserable, muddled, cheerful, bitter, exhausted, anxious, ready to fight, good natured, gloomy, desperate, sluggish, rebellious, helpless, weary, bewildered, alert, deceived, furious, efficient, trusting, full of pep, bad-tempered, worthless, forgetful, carefree, terrified, guilty, vigorous, uncertain about things, and bushed.
