# Supplementary material for: High-Dose Intravenous Vitamin C Combined with Cytotoxic Chemotherapy in Patients with Advanced Cancer: A Phase I-II Clinical Trial
Source: PLoS One. 2015 Apr 7;10(4):e0120228. doi: 10.1371/journal.pone.0120228 (PMC4388666; doi:10.1371/journal.pone.0120228)

Text S1 Individual values for vitamin C and oxalic acid concentrations and individual plasma vitamin C time-course profiles

Individual data points for Table 3 (Pharmacokinetic profiles before and after chemotherapy)

Before chemotherapy

Parameter	Patient 1	Patient 2	Patient 3	Patient 4	Patient 5	Patient 6	Patient 7	Patient 8	Patient 9	Patient 10	Patient 11	Patient 12
Vit C dose infused (g)	36	50	44	46	41	45	45	43	43	45	35	41
Vit C dose (mmol)	204	284	250	261	233	256	256	244	244	256	199	233
Baseline plasma conc (µmol/L)	47	9.5	35	29	84	28	47	292	36	56	38	96
Plasma conc end of infusion (mmol/L)	13.5	15.0	13.2	13.9	19.6	13.6	14.9	10.9	15.1	12.7	14.2	10.8
Plasma conc 4h later (mmol/L)	3.1	3.1	4.0	2.3	3.5	1.7	4.2	3.8	7.0	5.1	5.4	2.1
Vit excretion by end of infusion (mmol)	51.4	22.8	69.9	100	55.1	39.4	43.5	17.5	48.5	38.4	53.2	76.6
% of dose	25	8	28	38	24	15	17	7	20	15	27	33
Total excretion by 4h after end of infusion (mmol)	157	49.9	179	225	185	117	142	115	146	162	102	214
% of dose	77	18	72	86	79	46	56	47	60	63	51	92
Oxalic acid excretion by end of infusion (mg)	14.07	5.98	28.93	18.04	6.78	8.08	6.00	6.55	11.98	6.31	7.37	10.65
Total excretion by 4h after end of infusion (mg)	52.51	13.95	87.01	59.69	32.63	43.23	23.00	43.25	35.94	30.63	22.54	43.73
% of vitamin C dose	0.29	0.05	0.39	0.25	0.16	0.19	0.10	0.20	0.16	0.13	0.13	0.21

After chemotherapy

Parameter	Patient 1	Patient 2	Patient 3	Patient 4	Patient 5	Patient 6	Patient 7	Patient 8	Patient 9	Patient 10	Patient 11	Patient 12
Vit C dose infused (g)	36	50	44	46	41	45	45	43	43	45	35	41
Vit C dose (mmol)	204	284	250	261	233	256	256	244	244	256	199	233
Baseline plasma conc (µmol/L)	69	49	74	81	138	67	130	173	429	169	103	97
Plasma conc end of infusion (mmol/L)	13.2	12.4	15.7	13.5	18.4	11.3	13.6	9.3	14.9	12.7	13.3	13.5
Plasma conc 4h later (mmol/L)	2.9	2.9	4.3	3.4	2.3	1.7	4.5	3.6	7.1	4.3	3.5	3.2
Vit excretion by end of infusion (mmol)	55.2	13.8	46.3	42.7	71.5	24.3	29.6	25.7	44.7	29.7	31.7	55.7
% of dose	27	5	19	16	31	10	12	11	18	12	16	24
Total excretion by 4h after end of infusion (mmol)	149	48.8	176	169	144	87.0	172	119	158	156	89.4	124
% of dose	73	17	70	65	62	34	67	49	65	61	45	53
Oxalic acid excretion by end of infusion (mg)	34.10	3.41	27.01	10.07	8.73	10.82	3.94	9.76	18.82	5.88	6.12	8.70
Total excretion by 4h after end of infusion (mg)	66.81	13.48	79.00	53.48	22.75	51.09	29.58	38.85	55.53	29.20	21.61	29.78
% of vitamin C dose	0.36	0.05	0.35	0.23	0.11	0.22	0.13	0.18	0.25	0.13	0.12	0.14

Individual data points for Fig 2 (Plasma vit C concentration profiles µmol/L before and after chemotherapy)

Before chemotherapy

Time (hours)	Patient 1	Patient 2	Patient 3	Patient 4	Patient 5	Patient 6	Patient 7	Patient 8	Patient 9	Patient 10	Patient 11	Patient 12
0	47	10	35	29	84	28	47	292	36	56	38	96
0.5	6576	6297	5097	7370	9791	6093	7110	4814	7344	7725	7669	6138
1	9393	11044	9389	11209	16490	10645	11491	8321	12190	9910	11395	9550
1.25	11176	11905	11132	11912	17310	11826	12618	9534	13080	10709	12569	10736
1.5	13534	14947	13164	13926	19596	13635	14851	10876	15135	12679	14230	10828
1.75	11101	12142	12960	11760	13844	11519	12297	9065	13970	11363	13223	10367
2	10770	10902	12266	10500	12044	9395	11103	8542	12917	10711	12422	9117
2.25	8961	8873	11208	8956	10235	8462	8394	7930	12907	10183	11768	8138
2.5	8167	8280	10529	8517	10159	7570	8765	7528	12059	9509	10498	7221
3	7588	7033	8379	6426	7280	6297	7113	6826	11547	8594	9761	5763
3.5	5931	6318	7617	4876	6033	5166	7227	6306	10711	7494	9022	4657
4	4114	5187	6443	3805	5120	4330	5863	5625	9296	6156	7433	5760*
4.5	3726	4369	5611	3115	4630	3003	5785	4656	7712	5786	6738	2733
5	3681	3924	4721	3212	4018	2505	4926	4374	7584	5262	6022	2276
5.5	3092	3120	3960	2311	3482	1692	4150	3820	6972	5101	5426	2047

After chemotherapy

Time (hours)	Patient 1	Patient 2	Patient 3	Patient 4	Patient 5	Patient 6	Patient 7	Patient 8	Patient 9	Patient 10	Patient 11	Patient 12
0	69	49	74	81	138	67	130	173	429	169	103	97
0.5	7281	4633	5939	6462	6330*	4868	5169	3843	7263	7857	7260	9068
1	10397	8454	10419	11213	16898	9375	9845	6625	12367	10575	11547	11374
1.25	11525	11435	14055	11916	18370	10963	11811	8190	13655	11659	12226	13204
1.5	12226	11501	15729	13484	18041	11293	13579	9283	14889	12701	13308	13445
1.75	13228	12352	14083	11975	14489	9668	11730	8223	13788	11278	11810	11774
2	9776	11047	12407	11513	12355	8843	10685	7558	13407	10284	11021	9910
2.25	7942	8624	11833	10494	10710	7554	9839	6556	12545	8817	10311	9415
2.5	7089	7558	10399	9435	9585	6733	8805	6556	12310	8859	9953	8254
3	6246	6609	9340	6791	7473	5507	7687	5764	10958	7081	8741	6294
3.5	5214	5266	7812	6026	5004	4466	6151	5310	9934	6917	7744	5430
4	3800	4473	6888	4835	4096	3688	5605	4698	9296	5853	6222	4712
4.5	3454	3788	6095	3705	3552	2872	5036	3851	8690	5596	5372	3675
5	3345	3316	4904	2668	2901	2216	3404	4468	7552	4557	4283	2845
5.5	2900	2898	4327	3384	2299	1707	4455	3629	7089	4301	3515	3161

* 2 missing values interpolated from averages of all other values for that time.

Individual data for Fig 2 statement of AUC (area under the curve) in mmol.h/L before and after chemotherapy

Patient	Before chemo	After chemo
1	36.81	35.26
2	39.23	34.25
3	42.39	46.02
4	36.49	38.25
5	47.12	44.04
6	34.52	30.00
7	42.22	38.36
8	33.15	28.26
9	54.75	52.69
10	42.68	40.09
11	48.74	42.88
12	31.23	38.09

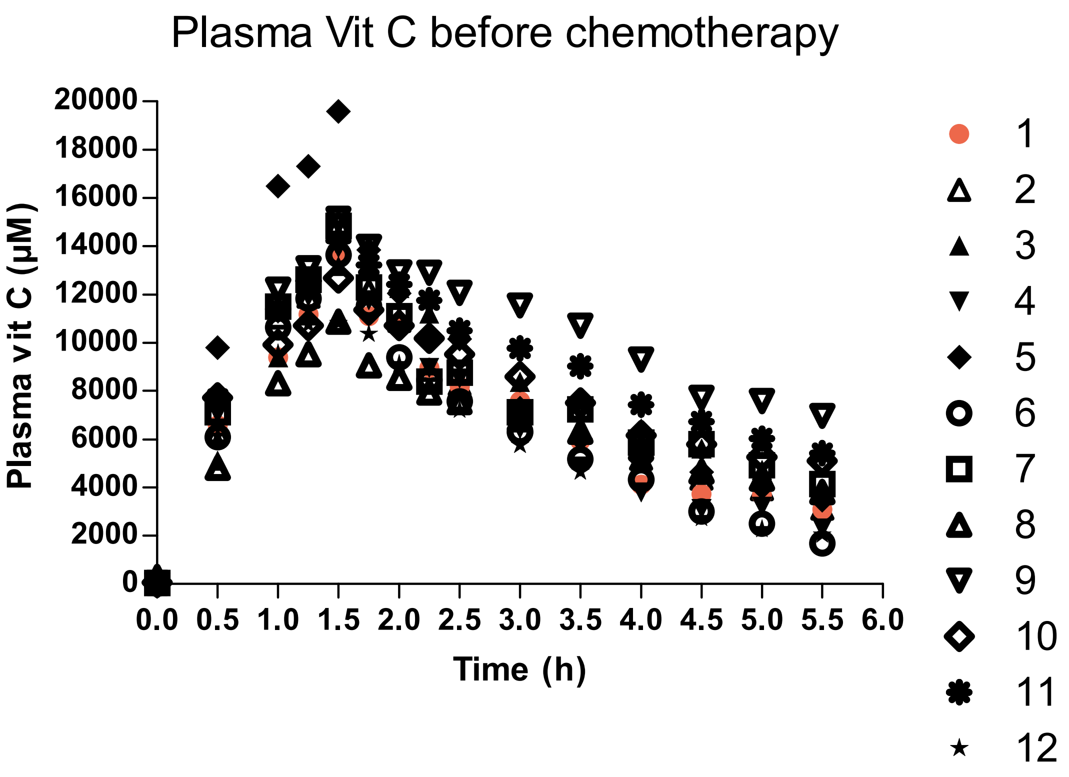
Plasma Vit C before chemotherapy
Plasma vit C (µM)
20000
18000
16000
14000
12000
10000
8000
6000
4000
2000
0
0.0 0.5 1.0 1.5 2.0 2.5 3.0 3.5 4.0 4.5 5.0 5.5 6.0
Time (h)
1
2
3
4
5
6
7
8
9
10
11
12

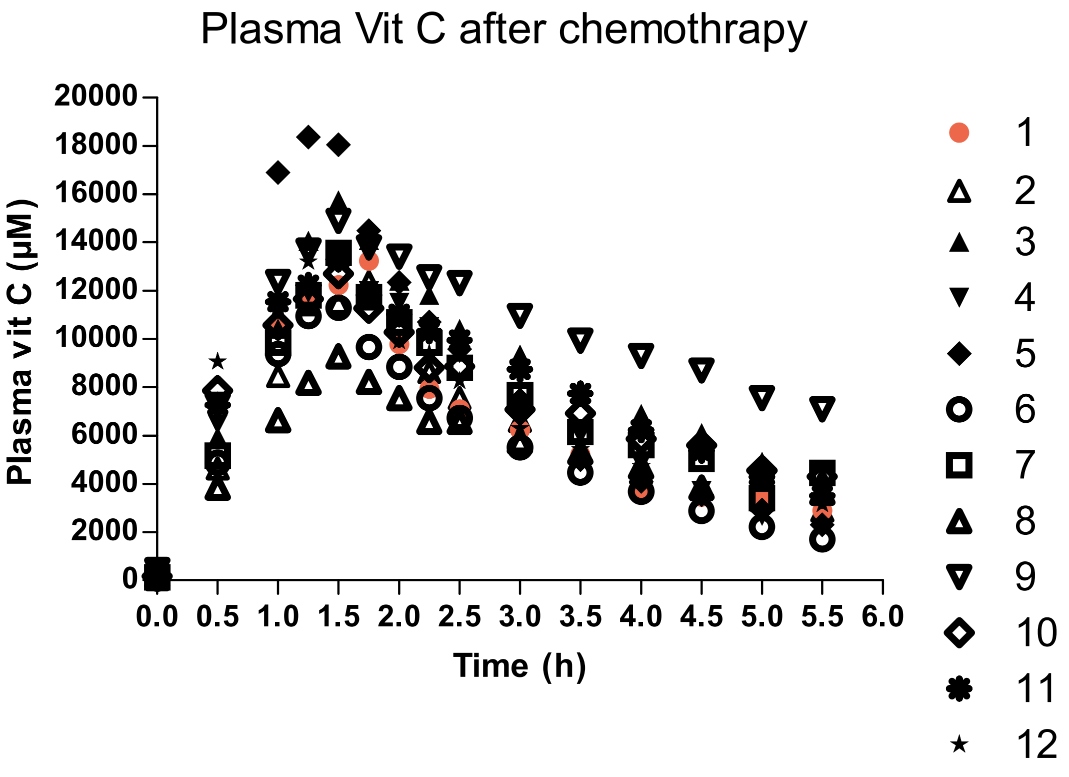
Plasma Vit C after chemothrapy
Plasma vit C (µM)
20000
18000
16000
14000
12000
10000
8000
6000
4000
2000
0
0.0 0.5 1.0 1.5 2.0 2.5 3.0 3.5 4.0 4.5 5.0 5.5 6.0
Time (h)
1
2
3
4
5
6
7
8
9
10
11
12

Plasma vit C before and after chemotherapy

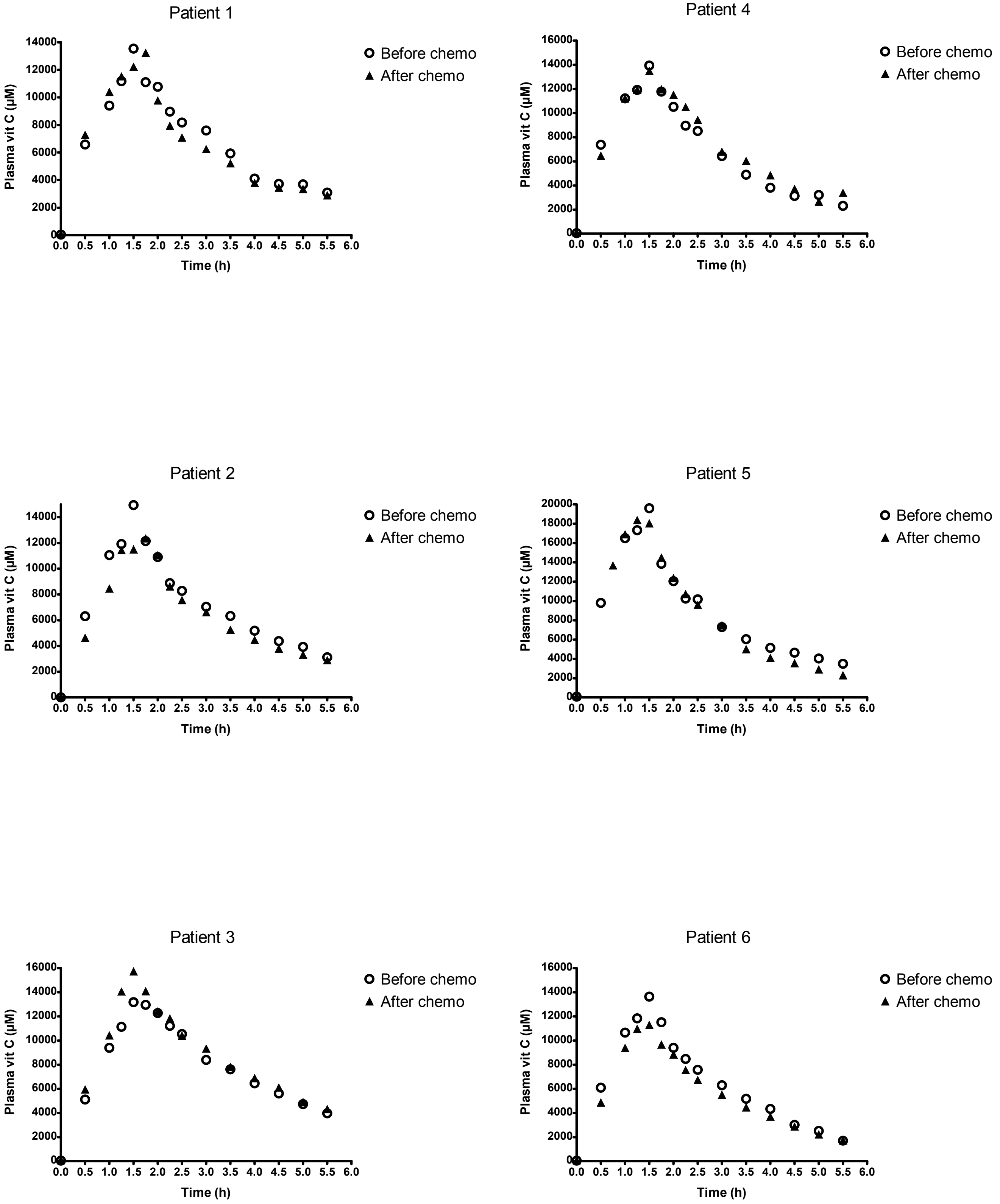

Plasma vit C before and after chemotherapy

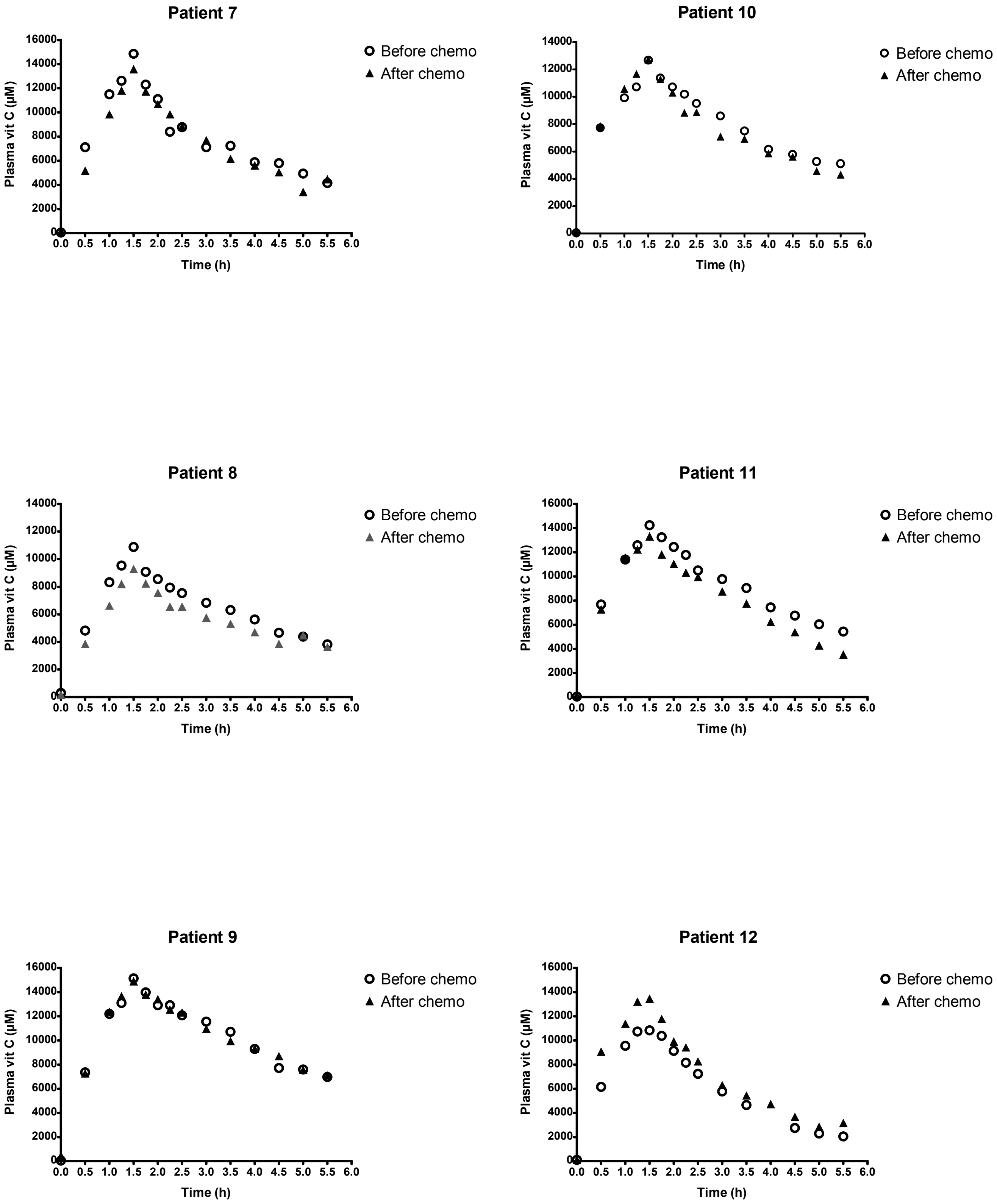

Supplement: S1 Text — (PDF) [file pone.0120228.s003.pdf]
