# Supplementary material for: High-Dose Intravenous Vitamin C Combined with Cytotoxic Chemotherapy in Patients with Advanced Cancer: A Phase I-II Clinical Trial
Source: PLoS One. 2015 Apr 7;10(4):e0120228. doi: 10.1371/journal.pone.0120228 (PMC4388666; doi:10.1371/journal.pone.0120228)
Supplement: S2 Text — (PDF) [file pone.0120228.s004.pdf]

Text S2 Detailed case information

(Reviewed November 3, 2014)

Patient 1
(May 2, 2013, June 28, 2012, May 2, 2014)
First IVC dose March 13, 2009
Last day of IVC June 8, 2009: Duration of therapy 85 days
Duration of known stable disease based on CT scan of April 29, 2014: March 13-April 29, 2009 = 46 days. This previously healthy 72 year old woman was discovered to have a RUL lung lesion on routine CXR in December 2008. Diagnosis was on the basis of abnormal cells in a bronchial washing specimens and biopsy of a lesion involving the left 5th rib, which indicated adenocarcinoma, stage IV. A Bone scan in December 2008 indicated increased radiotracer uptake in the anterior and lateral left 5th rib in keeping with a metastatic disease. On January 22, 2009 PET scan disclosed increased metabolism in the RUL lesion as well as increased metabolism in right hilar, pre-carinal and inferior paratracheal lymph nodes and the left anterior 5th rib. The staging CT scan on Feb 29, 2009 showed a 3.9 ill-defined mass in the posterior segment of the RUL, two lesions (0.8 and 0.4 cm) in LUL, 1.1 cm azygos node and 1 cm pre-carinal node, and 7 X 2 cm sclerotic-lytic lesion of the anterior and lateral left 5th rib. She enrolled in the study, with the first vitamin C infusion on March 13, 2009. The chemotherapy was Carboplatin plus taxotere 3 weeks apart on Tuesdays, with IV C 90 grams infused on the Monday, Wednesday and Friday of the week when chemotherapy was administered and on any two days at least 1 day apart during the subsequent two weeks without chemotherapy. (Chemo March 17, April 7, 2009). After the second cycle, a CT scan on April 29 indicated no change in any of the lung masses or the left rib lesion. After a further 2 cycles (chemotherapy on April 30 and May 21, 2009) a CT scan on June 3, 2009 reported no significant change in the primary RUL lesion or other target lesions. A new nodule of unstated size was noted in the posterior right apex. Despite the lack of change in the target lesions, the appearance of a new nodule was considered evidence of progressive disease. The protocol was terminated. Adverse events experienced by the patient included nausea, hypomagnesemia, and cytopenia as are common with this chemotherapy. There were no adverse events of any kind attributable to the IVC infusions. During the course of the study the patient expressed the view that she felt better during the vitamin C infusions and would have continued them. Summary: This patient with stage IV NSCL cancer experienced no progression after 2 cycles of chemotherapy and a small progression after 4 leading to termination of the protocol by her attending oncologist. Patients with this diagnosis have a 75% likelihood of progression with this chemotherapy give alone. However, it is impossible to determine from this study whether the lack of progression was attributable to the chemotherapy, to the natural history of the disease, or to the combination of chemotherapy and IVC. It is reasonable to conclude that the addition of IVC to standard cytotoxic chemotherapy in a situation in which any objective benefit is unlikely, did not limit or counteract this chemotherapy.

Patient 2
(June 6, 2013)
Final IVC infusion was 12 Feb 2010, total duration 34 days. This 57 year old man started IVC on January 10 at age 57. Rectosigmoid carcinoma was diagnosed 2006, low anterior resection Oct 2006 followed by adjuvant chemotherapy metastatic to liver, lungs and abdominal and pelvic lymph nodes. Therapy: Nov 21, 2006: xeloda, Dec 15, 2006: oxaliplatin plus xelodada 6 cycles. June 25, 2008, oxaliplatin, CPT-11, avastin for 10 cycles. March 30, 2009, CPT-11, 5FU, avastin for 25 cyles. He signed the consent form 22 Dec 2009. CT scan Dec 22, 2009: numerous lung nodules thru-out lung parenchyma, progressive since previous scan, multiple hypodensities throughout entire liver, retroperitoneal and paraaortic and retroperitoneal lymph nodes adjacent to the common iliac artery, no hydroureter or hydronephrosis. The treatment on-protocol was IVC 124 g per infusion plus CPT-11 plus iv 5FU and leucovorin plus avastin every two weeks. IVC was given 3 times during the week of chemotherapy and 2 / during the week off chemotherapy. He received this therapy from Jan 10 to February 15, a total of 6 weeks, when he developed bilateral leg swelling. A CT scan showed progression despite the new chemo and iv C. Protocol was dc. He was admitted to hosp Feb 19, 2010 with PICC line infection and treated, and DC. Protocol was ended. Patient continued to be treated in palliative care. He died 11 months later on Dec 27, 2010. (CT scan on Feb 18, 2010, 4 weeks after starting iv C, showed increased size of a LLL lung nodule, multiple new lesions in liver consistent with mets, progression of pelvic and retroperitoneal adenopathy with left common iliac vein external compression, and moderate left hydroureter and hydronephrosis.)

Patient 3
(June 21, 2013 and June 26-8 2013)
Protocol day one: 7 January 2010. Last IVC day: March 26, 2010. Duration of IVC 72 days. This 68 year old man was found to have a well differentiated invasive adenocarcinoma of the colon T4 (N1 m1 2 out of 34 lymph nodes positive) and shortly afterwards underwent a right hemicolectomy in January 2007. He received 7 cycles of Xeloda, Oxaliplatin and Avastin between March and August of the same year. Cancer progression was documented in November 2007. He then received 6 cycles of Folfiri chemotherapy between February and April 2008. A CT scan revealed progression. Chemotherapy with 3 week cycles of IROX (irinotecan = cp5-11 plus oxaliplatin) was administered between Aug and November 2009 until Nov 29, 2009, but the patient developed a severe allergic skin reaction to oxaliplatin during the second cycle. In December 2009 the patient was enrolled in the IVC clinical trial, the chemotherapy regimen being Folfiri. The patient started Vitamin C trial January 14, 2010, following a baseline CT scan (Dec 22, 2009) that showed a hypodensity in the right lobe of lung measuring 2.5 cm and multiple abdominal soft tissue implants, the largest measuring 2.9 x 2.53 cm in the splenic flexure. The patient received a total of 5 two-week chemotherapy (Folfiri) cycles (January 19, February 2, 16, March 2, 16, 2010) He received 110.0 g of Vit C, 3 doses on the week of chemotherapy and 2 doses during the week off chemotherapy. Unlike other patients he did not experience an increase in energy or well being during or after the iv c infusions. After the fifth cycle of cycle, a CT scan on March 18, 2010 showed progression of all the intra-abdominal implants, and the protocol was discontinued. The patient died July 24, 2010.
Summary: this patient with widely metastatic colorectal cancer entered the vitamin C protocol after his cancer progressed despite 4 previous chemotherapy regimens. He received IVC between

January 19 and March 18, a total of 2 months, at which time but progression was documented and the protocol was discontinued. The patient did not experience any side effects or toxicity attributable to the iv C therapy. Unlike other patients he did not experience an increase in energy or well being during or after the iv c infusions.

Patient 4
(June 28, 2013)
Protocol day 1 was 19 Feb 2010. Final IVC was April 6, 2010, total duration of treatment 41 days. Pt was diagnosed with rectal cancer in 2002, with surgical resection and chemoradiation therapy, with known residual disease after surgery. The disease recurred in 2007, both locally and as a localized presacral tumour mass, with increasing CEA concentration. Patient was treated with FOLFIRI and transferred to care of her oncologist for second line treatment. In Jan 2009 started therapy with Avastin plus oxalaplatin. Intolerance to XELOX so switched to IROX, until April 2009, when chemotherapy was stopped. Local brachytherapy in 2009 for tumour invasion of the cervix. CT on Jan 19, 2010: Fluid in endometrial canal, lymph nodes 18 X 10 mm right pelvic wall, tiny nodule 4 mm in anterior segment of the RUL. Cervix infiltrated by tumour.
Jan 19, 3010, baseline CT abdomen only: bladder thickening fluid filled endometrium. Started IVC protocol 19 February 2010 with Xeloda given in a 14 day out of 21 cycle concurrently, with ivc 3 per week for 2 weeks and twice per week on the week off Xeloda. CT April 22, 201 previous RUL nodule increased in size. D/C protocol April 6 due to s/e of vitamin C
March 1, 2010. Patient said the IVC gave her a sense of increased energy. Complained of fullness, bladder urgency and pressure LLQ in suprapubic area, with continual mild urinary urgency. Was self-catheterizing March 5, 2010. March 29, 2010 vomiting toward end of iv C infusions, while drinking fruit juices, April 5 vomited at end of IVC infusion. Ap 18, 2010 note: Withdrew protocol on Ap 6, 2010 because of vitamin C induced vomiting even at the late stage of infusion with a very low dose of 0.9 g/kg. Her dose in study was 115 g (wt 77 kg). Her dose Xeloda 1000 mg BID X 14 days q 21 d cycle. She subsequently died on August 1, 2010 at the age of 53.

Patient 5
(June 28, 2013)
Start day of protocol 3 April 2010; Last IVC June 11, 2010. Duration of IVC 62 days.
Dose 103 g. 58 year old woman who was diagnosed with rectal cancer in October 2007, treated with hemicolectomy and chemoradiation therapy. The disease recurred in 2008, for which a further surgical resection was carried out in December 2008. The patient had a pulmonary embolism in February 2009. In March 2009 lung and liver metastases were detected. She was treated with FOLFOX and AVASTIN for 4 months followed by FOLFIRI and AVASTIN, but the disease progressed despite these treatments. The patient was begun on the vitamin C protocol with concurrent Xeloda given as 2 weeks of therapy followed by one week rest. During the two weeks of Xeloda IVC was administered three times per week, and during the one week of rest given two times per week. The first dose of Xeloda was April 12, 2010. The baseline CT scan don April 8, 2010 showed bilateral pulmonary nodules, the largest 4.2 C 3 cm, and 2 liver lesions, the largest 2 X 1.7 cm. The follow up CT scan on June 3, 2010 showed increase in size of the lung and liver nodules. Disease progression was diagnosed and the protocol was discontinued. A note on drug regimens: Currently FOLFOX (5-FU is a 46-h infusion, plus folinic acid and oxalipoatin), or XELOX (prodrug capecitabine in place of 5-FU) or FOLFIRI (replaces

oxaliplatin with the topisomearase inhibitor irinotecan); all are accepted first-line treatments, combined with the anti-angiogenic agent bevacizumab = Avastin in the case of metastatic CR cancer. Avastin is commonly used as first-line treatment in metastatic CRC in combination with FOLFOX or FOLFIRI.

Patient 6
(July 2, 2013 updated May 2, 2014)
Last IVC August 24, 2010, Duration of IVC: 114 days. Date of last stable CT = June 22, 2010. So duration of known stable disease 43 days This 53 year old man was found to have rectal cancer in September 2008, and at surgery in October, when a left hemicolectomy was performed, he was found to have a moderately differentiated invasive adenocarcinoma with metastases to the lymph nodes, liver and lungs. As documented in a chart note on April 13, 2010, the cancer progressed despite two different chemotherapy regimens (FOLFOX and Avastin, and Folfiri and Xeloda). (FOLFIRI consists of Leucovorin, 5-Fluorouracil (5FU), and irinotecan and is approved for use in patients with advanced and metastatic colorectal cancer). He was started on IROX (oxaliplatin plus irinotecan) on April 13 but the oxaliplatin had to be discontinued during the second cycle of this regimen after a severe allergic reaction. The patient was enrolled in the IVC study: IVC plus CPT 11 (irinotecan) in a 3 week cycle, receiving the chemotherapy in April 13, May 4, May 25, June 15, July 6 and July 27. IVC was given as an add-on to continuing chemotherapy 3 days during the week of chemotherapy and 2 days on the non-chemotherapy weeks. The first therapeutic dose of IVC was given on May 3, 2010 (irinotecan was given May 4, 25, etc.) His study baseline CT scan on April 27, 2010 showed multiple metastatic lesions in the lungs, liver and abdominal lymph nodes. By July 13, 2010 he received 4 cycles (each 3 weeks) of CPT 11. A CT scan on June 22, 2010 showed that the liver lesions had increased slightly in size from 2 months earlier, as well as increasing size of peripancreatic and periportal lymph nodes, and an increase in a left lung lesion, but less than 20%, and hence considered stable disease; the protocol continued. A third CT scan on August 8, 2010 demonstrated increased in the sizes of multiple lung metastases and new brain metastases, as well as progression of peritoneal implants and liver metastases. Therefore the protocol was discontinued.

Patient 7
(July 16, 2013)
Protocol day one PK1 was 4 June, 2010. The last stable CT was July 27, 2010. Duration of known stable disease 53 days. Final IVC dose was Sept 29, 2010 (114 days). 76 year old man with metastatic colo-rectal cancer, met to liver and lungs. The cancer diagnosis had been made 3 years earlier, in 2007, age 73, treated first with Xeloda for 4 cycles, then starting in March 2008 with oxaliplatin, avastin and avastin for 4 cycles, then June 2008 with CPT-11/avastin for 10 cycles, then on Sept 24 23009 with DPT11/avastin/xeloda for 11 two-week cycles until March 27, 2010. June 4, 2011 started IVC, First full dose on June 7, 2010. Chemo was given June 8, 29, etc. in 3 week cycles of CPT-11 and Avastin, as well as Xeloda given 2 of every 3 weeks. The protocol was such that IVC was given 3 times on the week of chemo and 2/week the other 2 weeks. During the full dose (112 g), and unlike other patients, this patient experienced epigastric discomfort and a fluttery feeling, bordering on nausea, during the final minutes of the IVC infusion. He felt fatigue, haziness which persisted for a day following the infusion. CT scan of May 27, 2010: liver met 54 x 75 mm and multiple pulmonary nodules; CT scan of July 27, 2010, stable disease. Therefore protocol continued. IVC and chemotherapy continued until Sept 29 (for

IVC) and Sept 24 (for chemo), 2010. However the CT scan of Sept 30, 2010, increase in dimensions the liver metastasis to 77 X 88 cm. Therefore the protocol was terminated. The patient subsequently was treated with Vectibix (because of normal K-ras gene) and had an impressive biochemical and clinical remission. July 10, 2013: CEA May 12, 2010: 359, May 27 = 359, June 8 = 539 June 28 = 666 July 17 = 661 Aug 30 = 654.

Patient 8
(June 27, 2013)
protocol day one, 14 June 2010. Last IVC was 30 June 2010, duration of therapy 12 days. This man had a history of diabetes, coronary artery disease (MI in 2004), and atrial fibrillation. In 2008 he was found to have a high grade bladder carcinoma. A radical cystectomy and ileal conduit was created (September 3, 2009). There was venous, perineural invasion and direct extravesicular extension and regional lymph node involvement (PT4a pN1 pMx). Chemotherapy was given in January 28, 2010 through April 2010 (6 cycles) as 3-week cycles of cisplatin and Gemzar. A CT scan in June 4, 2010 showed bone metastses to left iliac bone extending into the soft tissues (4.4 X 3.1 cm) and posteriorly, and a smaller metastatic lesion in the acetabulum-ischium. IVC protocol began. June 18, 2010. The full therapeutic dose of IVC was given June 21, 23, June 28 and June 30, a total of 4 doses. Chemotherapy (Gemzar and cisplatin) was administered June 22, 2010. However on July 2, 2013 the patient became acutely ill and was admitted to hospital with fever and confusion, and found to have gram negative bacterial sepsis. The IVC protocol was terminated. The patient responded to antibiotic therapy and was discharged. He subsequently died Oct 18, 2010 in the hospital's palliative care unit. In summary, this patient with metastatic bladder carcinoma received only 4 doses of iVC before the protocol had to be discontinued because of a hospital admission for bacterial sepsis. It is impossible to make any conclusion about the vitamin C therapy in this patient.

Patient 9
(May 2, 2013, May 28, 2013)
Protocol Day ONE 25 Feb 2011; final IVC May 18, 2011. Duration of IVC: 83 days. Cancer of the left ovary with peritoneal involvement and ascites was diagnosed in April, 2008. The patient received neo-adjuvant chemotherapy (3 cycles of carboplatin and paclitaxel) followed by debulking surgery with residual disease remaining. In July 2008, a hysterectomy, oophorectomy and debulking of omentum was carried out. Omental biopsy showed mixed endometrioid and papillary serous adenocarcinoma (type II), Grade IIIC. The patient received post-operative chemotherapy with the same drugs. A CT scan in September 2008 indicated marked diminution of the main tumour mass in the lower abdomen and pelvis by comparison to the pre-operative CT scan, presumably because of the debulking surgery. Chart notations documented only a poor clinical response to postoperative chemotherapy. A pulmonary embolism occurred in September 2008. Chemotherapy continued over the following 2 years using doxorubicin, then taxol. Progressive disease was documented in January 2011 despite weekly taxol (documented June Sept 12, 19, 26, 29, Oct 6, Nov 15, Dec 1, 8 and 15) then carboplain Dec 22 and January 19, 2011. The patient was referred for the IVC protocol with the plan to continue previously ineffective carboplatin at roughly 3 week intervals with IVC therapy added. The protocol started Feb 25, 2011. The therapeutic vitamin C dose of 108 g was infused over a period of 2 hours. Carboplatin was given on Tuesdays. (March 1, 29, and April 27 with iv C given MWF the week of carboplatin and on any 2 days one or more days apart during non-chemotherapy weeks.)

When the protocol started the patient had ECOG 0. The physical exam disclosed a large firm, immobile lower abdominal mass. CT scan Feb 24 showed multiple pulmonary nodules, more in the right lung field. There was a large abdominal-pelvic mass with ascites, and nodularity indicative of peritoneal carcinomatosis. The large soft tissue density involved the anterior wall hernia, and there were implants within the hernia, as well as soft tissue densisty involving the deep pelvis lateral to the distal sigmoid colon. The patient experienced adverse effects of shakiness and thirst during the IVC infusions. These were mitigated by slowing the rate so that the dose was infused over 2.5 hours. The patient received a total of 3 cycles of carboplatin (March 1, 29, and April 27, 2011). Physical examination in early May suggested increased bulk of the lower abdominal mass and peri-umbilical bruising. A CT scan on May 18, 2011 showed a large cystic lesion with capsule in the abdomen measuring up to 19 X 3.3 cm. This large lobulated septated cystic mass, with associated ascites, was increased in size from the CT scan done on February 24. On the basis of this CT scan, the patient was determined to have documented progressive disease, and the protocol was terminated. Summary: This 65 year old woman with advanced multiply chemotherapy treated and chemotherapy resistant ovarian cancer received IVC in addition to 3 cycles of previously ineffective carboplatin administration. There was no indication of benefit.

Patient 10
(June 6, 2013, May 2, 2014)
Protocol day 1was March 3, 2011. The last IVC dose was on Sept 16, 2011, duration of IVC 193 days. The last stable CT scan was on May 25, 2011: 84 days of known stable disease. This woman was found, in 2007, at age 43 to have poorly differentiated adenocarcinoma of the cervix stage II B. She received 6 cycles of cisplatin as well as radiotherapy, and brachytherapy, but no surgery, and experienced a clinical remission. Her disease recurred in 2009. She was told in 2009 that no more therapy was possible and referred for palliative care treatment with an expected survival of less than 1 year. She instead came to JGH in November 26, 2009 for a second opinion and was treated in our centre starting January 2010 with carboplatin-paclitaxel in 3 week cycles. She experienced a prompt and dramatic improvement, with virtual disappearance of bilateral leg edema due to groin or intrapelvic lymph node involvement by the cancer. After the third chemotherapy cycle she developed an allergy to carboplatin. Carboplatin desensitization was used to allow the completion of a total of 8 cycles by June 2010. By that time the patient could no longer tolerate the debility induced by her chemotherapy, so treatment was changed to weekly taxol. However, with this therapy her disease gradually worsened, the most disabling feature of which was increasing right leg edema. Thus despite a dramatic response to carboplatin plus taxol, its adverse effects became intolerable, and its replacement with weekly taxol was not satisfactory since with this treatment alone her cancer progressed. In January 2011 the patient requested alternative therapy, so she was started on iv Vitamin C plus taxol, the latter given only every 3rd week. After an injection of taxol in February 2011, she was enrolled in the vitamin C protocol (3 times on week of chemo and 2/week on weeks of no chemo) with injected taxol every 3 weeks (dates: March 8, 29 and April 19, 2011). She also took large doses (10,000 IU per day) of vitamin D. With this therapy her disease was stabilized as indicated by at CT scan 2 months into therapy. Despite the stabilization of her disease, the patient found the side effects even of taxol injections every 3 weeks to be intolerable, and chose to discontinue it, and requested to continue IVC. The iv C infusions (112 g) caused and cold and rumbling feeling inside, and thirst. Nevertheless she persisted with the infusions and after 4 weeks of infusions

she claimed to experience a pronounced increase in her energy level to normal, after each IVC infusion, the energy improvement lasting a few days. She specifically contrasted this feeling to the “bloated fatigued” feeling she was accustomed to have with her disease, and occurred despite that fact that the development of edema of both legs (worse in the right leg which was swollen) after each infusion, that took several days to resolve. Her noted shakiness, headache and thirst during each iv C infusion were no longer present when the infusion rate of the IVC was reduced. On August 24, 2011 (175 days of IVC) a CT scan showed an increase in the size of one of 3 signal tumor masses. Upon receiving this report the patient opted to stop further taxol, because she had chosen to value QOL. She wanted to continue the IVC, and this was done owing to her remarkably good QOL. Despite objective evidence of progression the patient continued to feel globally very well, better, she claimed than at any time in the previous 4 years. She reported that her right lower limb edema was decreased in size by about 50%, that she could walk 1 km/day, and she was attending the YMCA and swimming. Her mood and energy level were normal. She attributed these benefits to the IVC infusions, and continued to refuse to receive more chemotherapy. On September 7, 2011, the patient experienced twitching of the right arm and face, and a CT scan showed a left frontal cortical mass. At her request, all anti-cancer therapy treatment, including vitamin C, was stopped, and she was referred for hospice care. In summary, this 48 year old woman with advanced poorly differentiated cancer of the cervix that had progressed during treatment with taxol every 7 days appeared to stabilize with a regimen of IV C, taxol every 21 days and possibly high-dose vitamin D. During the course of therapy her quality of life improved dramatically. She directly attributed the improvement of energy and quality of life to the vitamin C injections, even though each injection itself was uncomfortable. (It is quite possible some of the improvement in quality of life was due to her being spared from the adverse effects of chemotherapy).

Patient 11
(May 29, 2013, May 2, 2014)
Day one of protocol was 22 July 2011, total duration 580 days. Date of last stable CT scan, August 21, 2012. Stable disease lasted from 22 July 2011 to August 21, 2012, 397 days. The cancer diagnosis was adenocarcinoma of ampula of Vater (cholangiocarcinoma). This patient received chemotherapy (gemcitabine iv q 3 week plus oral Xeloda), 5 cycles between 28 Jan and 22 April 2011. A biliary stent and indwelling cholecsystostomy tube were required. The IVC protocol started August 3, 2011, with 88 g infusions the week of chemo and 2/week on remaining 2 weeks of each cycle. Chemotherapy consisted of iv oxaliplatin and oral Xeloda in 3 week cycles. CT scan report of Sept 13, 2011 showed ERCP-placed biliary stent and a cholecystostomy tube, with moderate bile duct dilation. There were innumerable scattered hypodensities throughout the liver compatible with diffuse metatastic disease. The patient received this chemotherapy between August 2011 and November 2012, since the CT imaging, every 2 months, continued to show stable disease. Thus failure to progress lasted from August 3 2011 to November 20, 2012, a total of 17 months. For 17 months there was no progression of her extensive cancer during which ECOG status remained constant at 1. On that day there was a new 4 mm RML lung nodule, worsening bile cut dilation (11 cm to 17 mm) and although most liver metastatic nodules were stable, one of them had enlarged from 7 x 9 cm to 12 x 10 cm. On November 28, 2012, progression was noted. The treating oncologist requested to continue the patient’s IVC to allow for an alternative chemotherapy regimen to be contemplated. Between November and January 2013 no chemotherapy was given but IVC continued at the request of the

oncologist. On February 18, 2013 the IVC treatment protocol was discontinued because the treating oncologist was unable to justify any particular chemotherapy regimen. Approximately 4 days after that the patient fell at home and was found to have a large metatastic lesion of the C2 cervical vertebral body. She was admitted to hospital for palliative radiation and hospice care, passing away on March 4, 2013. In summary, this 54 year old woman developed biliary obstruction caused by an adenocarcinoma of the ampula of Vater (cholangiocarcinoma) that required urgent placement of a biliary stent and cholecystostomy tube. There were multiple liver metastases. Five three-week cycles of intravenous gemcitabine and plus oral xeloda failed to arrest progression of the cancer. The patient enrolled in the IVC trial with 3 week cycles of intravenous oxaliplatin and oral Xeloda. IVC (88 g) was given 3 times during week of intravenous chemo and 2/week during the other two weeks. The baseline CT scan showed innumerable scattered hypodensities throughout the liver compatible with diffuse metastatic disease. The patient remained on this protocol for 17 months with stable disease and unchanging functional status with CT scan monitoring every 2 months, at which time the CT scan indicated a small new lung nodule and worsening bile duct dilation. One of the many liver masses had now enlarged from 7 x 9 cm to 12 x 10 cm. It was concluded that that disease had resumed progressing. IVC continued to allow time to consider an alternative chemotherapy regimen, but no further chemotherapy could be scientifically justified. The patient continued to receive IVC for a further 3 months, without chemotherapy, but the protocol was finally terminated because the patient was no longer eligible. A few days after the protocol was ended the patient was found to have a large metatastic lesion involving the C2 cervical vertebral body. She was admitted for palliative radiation and hospice care.

Patient 12
(May 2, 2013)
Day one of protocol 19 August 2011. Final IVC Oct 4, 2011, 46 days. This was a 50 yr old woman with right breast cancer diagnosed in 2007. She has had received 4 cycles of neoadjuvant Herceptin and taxol chemotherapy and then underwent segmental resection February 28 2008. She then was referred to radiation therapy for radiotherapy. She received 50Gy in 25 fraction to the right breast and supraclavicular and axillary lymph nodes. Subsequently she was maintained on Tamoxifen until June 2009 where she developed a recurrence. She then received Navelbine and Herceptin which lasted from October 15, 2009 to December 29, 2009. In May 2010 the patient developed bone metastasis to the sternum. She was referred to a clinical trial with Xeloda and Lapatinib, then went on to Arimidex and then was switched to Aromasin. Her PET scan in September 2010 showed progression of metastasis in the internal mammary adrenal and periaortic lymph nodes sternal lesion and L3 vertebral body. The patient was then referred for evaluation at the US National Cancer Institute. November 24, 2011, the patient went to NIH and received experimental vaccine with Taxotere. The patient’s disease progressed and in August was referred for the IVC trial. She began IVC protocol August 19, 2011 in conjunction with oral chemotherapy Xeloda and Herceptin. She completed 2 cycles and decided she did not want to continue due to the large time commitment. Her last IVC dose was Oct 5, 2011. When protocol started her baseline physical exam showed no abnormalities with an ECOG of 0. Her baseline CT scan of August 8 2011 showed increasing pleural effusion, increasing right middle lobe atelectasis, and a precarinal mass measuring approximately 3 cm. Prior to starting the protocol, she experienced back pain and sleep disturbance both Grade 1 intensity and while on trial nausea and shortness of breath both at a Grade 1 level. This patient received 2 cycles of Vit C in

conjunction with Xeloda and Herceptin. Therapeutic doses (102 g) of IVC were given twice weekly on week 1 and 3 times per week during cycle week 2. Unfortunately the patient found the trial to be very time consuming and withdrew consent. She stopped treatment October 5, 2013

Patient 13
(May 27, June 6, 21, and 28, 2013)
Protocol day 1: 28 Oct 2011. Last day of IVC was 8 June 2012, duration of therapy 220 days. Feb 16, 2012 Stable disease from Oct 28, 2011 to Feb 16, 2012 = 112 days. This 63 yr old man was diagnosed at another centre in 2009 with a poorly differentiated epidermoid carcinoma of the head and neck, diagnosed in 2009 from biopsies of the right and left tonsils. The cancer involved the base of his skull, paranasal sinuses, sphenoid sinuses and chest. He was treated with radiotherapy and chemotherapy: 3 cycles of Carboplatin / 5-FU and radiation therapy between July 20 2009 and September 2 2009. January 19, 2010, the primary lesion had regressed and the patient felt normal. He attended a surveillance visit on July 14, 2011 at which his physician felt an abnormal nodule in his palate, and a biopsy confirmed invasive epidermal carcinoma. On July 21, 2011 his case was discussed by the tumor board at his hospital. It was the consensus of his physicians that he receive palliative chemotherapy. In August 2011, his nasopharynx showed a mass of approximately 5.8 cm, also invading the left maxillary, the soft and hard palate. Further therapy was not recommended. He came to our hospital in September 2013 for a second opinion and was enrolled in the IVC protocol on October 28, 2011. He received a therapeutic dose of 86 g of Vitamin C in conjunction with carboplatin and Taxol. His first iv C was Oct 26, 2011. He was treated with carboplatinum plus docetaxel in 3 week cycles given on a Tuesday with IVC given MWF on the week of chemo, then 2/week during the subsequent 2 weeks. His baseline CT scan on October 19, 2011 showed a large destructive mass in the left nasal cavity with destruction of the medial wall of the left maxillary sinus and nasal turbinates and extension into the sphenoid sinus with destruction of the floor of left orbit and minimal intra orbital extension with extension into the left maxillary bone, parapharyngeal soft tissues and left hard palate. He also had a metastatic lesion to the left upper lobe of lung measuring 2.5x 2.4 cm. November 25, 2011 he reported that over the previous 2 weeks there was definitely improved hearing in the left ear and his previously partially occluded left nasal passage now was free, allowing him to sleep normally for the first time. He was able to open his jaw more fully. He felt well. After three (3 week) cycles of IVC/chemo the CT scan done December 22, 2011, showed extensive tumor involving the left skull base, sphenoid, ethmoid, and maxillary sinuses, masticato space, posterior floor of orbit, and upper nasal fossa, not different from the scan done 2 months before (stable disease). The left upper lobe lung lesion measured 2.2 cm and was partially cavitated. The patient continued on trial and had 3 more cycles (3 week) of chemo and IVC. On February, 13, 2012 reported worsening left ear hearing acuity again but also decreased numbness in his left jaw. The CT scan of February 16, 2012 indicated that the locally invasive tumor in the left skull base had mildly decreased in size. The lung nodule was of diameter 2.2 cm and was partially cavitated. The RECIST diagnosis was stable disease. Despite the worsening hearing acuity he felt very well, was exercising, and on the dates March 17-29, 2012 he temporarily discontinued both chemotherapy and IVC in order to go on a vacation. Upon returning, he reported that during the final 2 days of the vacation, he experienced increasing feeling of pressure in his hard palate. A CT scan done on April 20, 2012 after his return to Montreal confirmed progression, with an increased size of all the lesions. It was decided to change the chemotherapy regimen to gemcitabine and oral Xeloda with continuing

IVC. This regimen was continued for 2 cycles but the CT scan on June 7 2012, showed heterogenous progression of all lesions including those in the lungs. On June 13, it was decided that patient stop therapy as it was no longer working. The patient died October 8, 2012. Summary: This patient had a hopeless prognosis but was given chemotherapy and IVC. He had a temporary disease stabilization and marked improvement in well being. Ultimately the tumor progression resumed, especially after he temporarily stopped chemo and IVC. It is impossible to determine from this single case whether he would have had a similar palliative benefit from chemotherapy alone, but there is no evidence that the vitamin C therapy prevented effective chemotherapy.

Patient 14
(July 4, 2013)
Protocol day 1 was 28 November 2011, final IVC was Dec 9, 2011, infusions for 11 days. This 53 yr old man was diagnosed with small cell carcinoma of the lung in 2008, treated in October 2008 with radiation therapy and cisplatin and etoposide chemotherapy, as well as radiation therapy to the brain for a small metastasis to the left brain. The tumor recurred within 3 years. On June 21, 2011 during a tumor board discussion, it was decided the patient could be considered either for weekly Taxol or Vit C clinical trial. Patient was enrolled in IVC trial November 25, 2011. The baseline CT scan showed a 3.1 X 1.8 cm mass in right upper lobe of the lung invading the right side of the mediastinum and superior vena cava. The protocol involved IVC given as 2 week taxol cycles with IVC given 3 times during the week of taxol/ IVC was given November 28, 30, Dec 3 (chemo, Dec 2), 2011, and again Dec 5 and Dec 9, 2011 for a total of 5 doses. The patient experienced dyspnea because of the cancer, but there was no increase in dyspnea or oxygen desaturation during the 96 gram vitamin C infusions. However, upon his arrival to the CRU on Dec 12, 2011 his dyspnea was worse so no IVC was administered and he was admitted to hospital. The protocol was terminated. Patient passed away January 11, 2012.

Tumor markers, FACT-G & POMs

<u>Patient #1:</u>
CEA: slightly higher than normal (8.2) with a tendency to lower levels after IVC (5).
CRP: remained normal (~2) before, during and after IVC.
CA125: Normal-high levels (~30) throughout IVC and after.
FACT-G: Fairly constant (~100).
POMs: Improved right after baseline (from -6 to -14) and remained constant.

<u>Patient #2:</u>
CEA: higher than normal at baseline (40-60), increasing somewhat during IVC, and increasing 20x after IVC, 10 months later.
CRP: High at baseline (148), lower but increasing during IVC, and normal 2 months later.
FACT-G: Fairly constant during IVC (~60) and decreasing to 46, 2 months post IVC.
POMs: some improvement from baseline (22) to last IVC (16) and worsening (35) 2 months post IVC.

Patient #3:
CEA: Higher than normal (~130) before and during IVC and more than doubles afterward (~200).
CRP: Normal before and during IVC (~3-4) but increased above 10 after IVC.
Fact-G: Fairly constant (~70)
POMs: Some improvement from baseline to end of IVC (2 to -8) with a worsening trend afterward (up to 3).

Patient #4:
CEA: High (~35) and no change during and after IVC.
CRP: High and variable (14-94) during IVC.
CA125: Normal (20) but only one measure on last IVC.
FACT-G: Fairly constant during IVC (~75).
POMs: Increasing from 9 to 22 during IVC.

Patient #5:
CEA: High at baseline (22), increasing during IVC (to 55), increasing even more afterward to 108 (~2 months later).
CRP: Fairly normal before and during IVC (~10), but increasing to 84 a month later.
CA 125: Normal (~20) before and during IVC, but increasing to 71 a month later.
CA19-9: Only one value before IVC and high (260).
FACT-G: Fairly constant (~80).
POMs: Worsening from -9 to 3 during IVC.

Patient #6:
CEA: High before IVC (~500) and increasing to 1226 at end of IVC.
CRP: High before IVC (~30) and increasing and variable during IVC (123, 60, 108).
FACT-G: Slight decrease from 76-66 during IVC.
POMs: Improvement from 13 to -1 during IVC.

Patient #7:
CEA: High before IVC (359), increased to 671 during IVC, then doubled to 1292 2 weeks after IVC.
CRP: Normal during IVC (~3) and slight increase to 8, 2 weeks after IVC.
FACT-G: Fairly constant (~70).
POMs: Fairly constant (~15), increasing to 25, 2 weeks after IVC.

Patient #8:
CEA: Somewhat higher than normal (~ 4-5) before and during IVC.
CRP: High before (35) and at beginning of IVC (54) but no other measurement.
CA125: Only one high (137) measurement during IVC.
FACT-G: Only one (77) measurement during IVC.
POMs: Only one (33) measurement during IVC.

Patient #9:
CEA: Normal (~1.5) during IVC.

CRP: High (32) and decreasing to 14-15 during IVC.
CA125: High (118) and increasing during IVC to ~ 200.
FACT-G: Only 2 measurements during IVC (~50).
POMs: Only 2 measurements during IVC (~37).

<u>Patient #10:</u>
CRP: Normal from 10 to ~5 during IVC.
CA125: High (230) and increasing during IVC to 1031.
FACT-G: Somewhat increasing from ~40 to 50 during IVC.
POMs: Up and down with no trend (between 16 and 42) during IVC.

<u>Patient #11:</u>
CRP: Normal (<10) for 13 months, then increasing to ~30 during last IVCs.
CEA: High (~30) and fairly constant throughout IVC.
CA125: Normal (12-20) except last measurement (65) last month of IVC.
CA19-9: Always normal-low (~1) while measured (first 11 months).
FACT-G: Fairly constant (~55).
POMs: Fairly variable with no trend (7-52).

<u>Patient #12:</u>
CEA: Normal (4-6) before IVC but increasing to ~30 during IVC and after.
CRP: Normal and increasing from <1 to 11 during IVC, then increasing to 20-25 after IVC.
CA125: High before IVC (~160) and increasing to 375 during IVC during IVC and to 784 thereafter.
CA15-3: High before IVC (~221) and increasing to 711 during IVC during IVC and to 1000 thereafter.
FACT-G: Only 2 measurements during IVC (~70).
POMs: Up and down during IVC (16, 30, 17) and somewhat increased after (36).

<u>Patient #13:</u>
CRP: Normal before IVC (8) and variable during IVC (from 3-19) and increased to 31 after.
FACT-G: Variable from 66 to 83 during IVC and lower (41) after.
POMs: Variable with relative improvement for first 5 months (6 to -1), then increasing to 29 last 3 months.

<u>Patient #14:</u>
CRP: High and constant (~30) before and during IVC.
FACT-G: Only one measurement during first IVC (84).
POMs: Only one measurement during first IVC (0)
